# ZDAM: a new deep learning model for bean leaf disease diagnosis

**DOI:** 10.3389/fpls.2026.1842022

**Published:** 2026-06-11

**Authors:** Jia Liu, Kaidi Yu, Hongyun Song, Jianyu Ma, Madineh Bijani, Longguo Wu, Laixiang Xu, Mohammad Nazir Ahmad, Peng Xu, Junmin Zhao

**Affiliations:** 1 School of Computer and Artificial Intelligence, Henan University of Urban Construction, Pingdingshan, China; 2 Faculty of Creative Media and Innovative Technology, Kuala Lumpur University of Science and Technology, Kuala Lumpur, Malaysia; 3 Department of Agroecology, Shahid Beheshti University, Tehran, Iran; 4 Faculty of Agriculture, Forestry and Ecology, Ningxia University, Yinchuan, China; 5 Institute of Visual Informatics, Universiti Kebangsaan Malaysia, Bangi, Malaysia; 6 Key Laboratory of Modern Agricultural Equipment of Jiangxi Province, Jiangxi Agricultural University, Nanchang, China

**Keywords:** attention mechanism, bean leaf, deep learning, disease identification, smart agriculture

## Abstract

**Introduction:**

Accurate disease diagnosis is crucial for enhancing agricultural productivity and reducing postharvest losses, directly impacting food quality and safety. Traditional detection methods often rely on extensive feature modeling and perform poorly in complex field environments.

**Methods:**

This study proposes a deep learning model called ZDAM, based on an improved ZFNet integrated with a dual attention mechanism. The classical ZFNet is first optimized to improve feature extraction efficiency. A combined channel and spatial attention mechanism is then incorporated to refine feature representation for disease identification in key crops. Finally, a residual module is added to boost accuracy.

**Results:**

Evaluated on a dataset of 11,903 bean leaf images covering healthy leaves and four disease types, including leaf mould, rust, mosaic, and white spot, the model achieves an average recognition accuracy of 99.02%, outperforming MobileMamba, Vision Transformer, and Chest- OMD.

**Discussion:**

This approach offers a scalable solution for automated disease monitoring, supporting postharvest quality preservation and sustainable crop production.

## 1 Introduction

In modern agricultural crop safety and quality, accurate identification of crop diseases, particularly in staple grains, is essential for maintaining crop health, enhancing yield, and improving product quality. Effective diagnosis directly influences the efficiency of disease monitoring, supports agricultural management decisions, and refines crop health assessments, thereby reducing postharvest losses and ensuring food safety. Current approaches to disease identification primarily depend on manual inspection or conventional image processing algorithms (Liang et al., 2025). These methods are often constrained by visual fatigue, subjective bias, and limited feature extraction capabilities (Mingle et al., 2024), falling short of the demands for rapid and precise production and postharvest handling in modern agriculture.

Accurate diagnosis of crop diseases such as rice depends on robust image-based feature extraction and classification algorithms (Gan et al., 2025; Lu et al., 2025). This task is challenged by natural field conditions: variable lighting and viewing angles alter leaf appearance, environmental factors damage leaf integrity (Zhai and Xu, 2023), and growth-stage morphological variations affect algorithmic consistency (Suonan et al., 2025). These combined complexities render automated disease identification a significant technical hurdle in practical agronomic and postharvest management (Zia et al., 2025).

Conventional plant disease identification methods typically rely on handcrafted image features such as color, texture, and morphology. It is combined with image processing techniques. For example, Jung et al. (2025) developed a support vector machine (SVM) classification model achieving 83.52% accuracy. Farhan et al. (2025) used high-throughput sequencing, network analysis, and machine learning to identify wheat leaf rust and obtained an accuracy of 90%. Feng et al. (2025) applied an SVM classifier for character segmentation and recognition, attaining 92.0% accuracy. Vasupalli et al. (2024) designed a plant leaf disease identification model based on an improved deep learning algorithm. Although such methods can perform basic disease identification, they require substantial manual design and optimization (Pei et al., 2025) and struggle in complex agricultural environments with diverse disease types, ultimately impacting horticultural product quality.

In recent years, deep learning has gained prominence in plant disease identification due to its powerful feature learning and adaptability. Convolutional neural networks (CNNs), as a core deep learning architecture (Wang et al., 2025), automatically extract high-level features from crop disease images through multiple convolution and pooling operations, enabling efficient identification. For instance, Yu et al. (2024) designed an RGB and infrared feature fusion segmentation network for plant pest and disease detection, achieving 94.0% accuracy. Belmonte-Sánchez et al. (2024) proposed a robust deep learning method for locating and classifying tomato leaf diseases, achieving 96% accuracy. Li et al. (2024) introduced an improved deep learning algorithm, TRG2P, for disease detection in rice, maize, and wheat, improving average accuracy by 16.17%. However, disease manifestations under field conditions, such as in rice paddies, are highly diverse (Olamofe et al., 2025), and factors such as complex backgrounds and variable lighting continue to challenge recognition performance, which is critical for pre-harvest monitoring that safeguards postharvest outcomes.

To address these issues, we propose a crop disease detection method based on an improved ZFNet integrated with a dual attention mechanism, with validation extending to key agronomic and horticultural species. ZFNet, a classical CNN architecture, has demonstrated strong performance in image classification. We optimized the classical ZFNet by simplifying modules and compressing channels, reducing model parameters while preserving the backbone network’s feature extraction capacity. A convolutional block attention module (CBAM) was embedded after convolutional and pooling layers. The CBAM captures long-range spatial dependencies via one-dimensional global average pooling along the height and width dimensions, generating channel attention weights that dynamically emphasize discriminative features. This design enhances recognition under common scenarios such as leaf self-occlusion or scattered lesions in crop leaves. It also strengthens local geometric and edge cues at higher semantic levels, reducing classification errors.

After upsampling the deepest feature maps and concatenating them with shallow features, we inserted a residual (Res) unit after each CBAM. This enhances the semantic representation of lesion areas and suppresses background interference through hierarchical context aggregation. It also refines feature expression for small lesions at higher resolutions. The fusion of Res units with multi-scale dilated convolution and CBAM further strengthens semantic discrimination of disease features, improving classification accuracy.

We enhanced the classic ZFNet by integrating channel and spatial attention mechanisms along with residual connections (Dai et al., 2022), enabling more effective extraction of key features relevant to crop diseases. This improves model robustness, recognition accuracy, and diagnostic efficiency in agronomic science and postharvest quality assurance applications. Therefore, we proposed a bean leaf disease classification model based on an improved ZFNet. First, we optimized the classic ZFNet by simplifying its modules and compressing its channels, reducing the number of parameters while preserving the backbone’s feature extraction capability. Second, after refining the convolutional and pooling layers, we embedded a Convolutional Block Attention Module (CBAM). This module captures long-range spatial dependencies via one-dimensional global average pooling along the height and width directions, generates channel attention weights, and enables the network to dynamically emphasize key features across different channels. In common scenarios such as leaf self-occlusion or scattered lesions, the model can more accurately locate lesion boundaries. Embedding CBAM in shallow layers amplifies local geometric structures and edge cues of lesions, while in deep layers, it enhances the semantic distinction between lesions and backgrounds, thereby reducing misclassifications and missed detections. Finally, after upsampling the deepest feature map and concatenating it with shallow features, we embedded residual units after each CBAM module. Through skip connections, this design adaptively enhances high-level semantic features in lesion areas and suppresses background interference during multi-source information fusion while maintaining smooth gradients. Placing Res units after CBAM integrates the semantic focusing ability of CBAM with the nonlinear transformation capability of residual learning, achieving more accurate localization of disease features. Thus, By integrating channel and spatial attention mechanisms with residual connections, this enhanced ZFNet more effectively extracts key features related to crop diseases, improving robustness, recognition accuracy, and diagnostic efficiency in agricultural science and post-harvest quality assurance applications.

The main contributions of this paper are as follows:

1. Simplification of classical ZFNet: By reducing structural layers and computational parameters, we improve inference speed and efficiency, yielding a lightweight model suitable for resource-limited devices.
2. Integration of dual attention: The simultaneous use of spatial and channel attention enhances focus on discriminative features, improving recognition accuracy under complex horticultural and field conditions.
3. Incorporation of residual modules: Skip connections mitigate gradient issues in deep network training, accelerate convergence, and enhance representational capacity.

For the first time, we incorporate CBAM and Res units at shallow and deep layers of ZFNet, enabling cooperative optimization of spatial localization and small-lesion enhancement. The Res unit is specifically integrated into the convolution-pooling structure based on visual characteristics of crop lesions, improving classification of small targets. Structural simplification balances robustness and deployment efficiency by controlling channel count and module depth, effectively managing computational cost while improving accuracy.

In summary, we design and validate a ZFNet-based optimized framework for high-precision classification of crop diseases under resource-constrained scenarios. This approach combines lightweight design with complementary attention mechanisms, customized for integration at critical network nodes. The proposed model has a parameter size of only 9.32 MB, enabling efficient deployment on edge devices such as unmanned aerial vehicles for real-time classification of crop leaf diseases, including in rice cultivation systems. This outcome holds significant practical value for advancing disease monitoring in agronomic and horticultural applications, including UAV-based scouting and mobile diagnosis, contributing to sustainable production and improved postharvest outcomes.

## 2 Proposed methods

### 2.1 Optimized ZFNet

**Figure 1:**
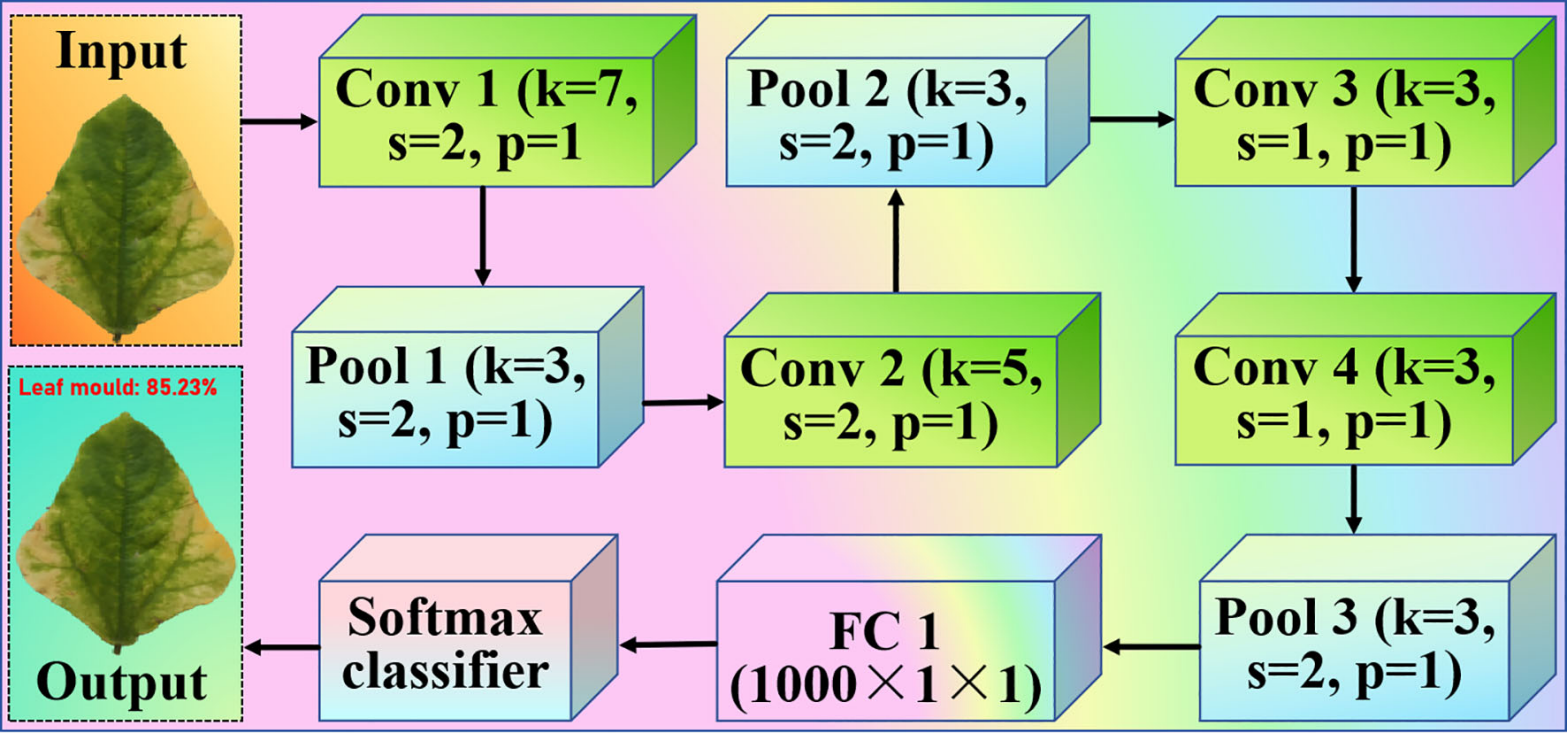
Improved ZFNet. The improved model is composed of five convolution layers, three pooling layers, one full connection layer, and one output layer.

Based on the data characteristics of bean disease imagery, this study enhances the classical ZFNet architecture to improve recognition performance. To optimize efficiency and generalizability, we remove one convolutional layer and one fully connected layer from the original structure. This simplification reduces model parameters and computational complexity, mitigates overfitting risks, and stabilizes gradient flow during training. The refined network comprises 5 convolutional layers, 3 pooling layers, 1 fully connected layer, and an output layer, as illustrated in **Figure 1**, where k, s, p, and n denote kernel size, stride, padding, and output categories, respectively. This lightweight design enhances suitability for deployment in resource-limited horticultural applications.

In the convolution layer, the feature maps from the previous layer participate in the convolution operation through the convolution kernel. The results are processed by an activation function to form a new layer of output feature maps. The output feature maps of each layer establish a convolution relationship with the input feature maps of the previous layer. It can be defined by Equation 1:

(1) $$x_{j}^{i}=f(\sum_{i\in M}w_{ji}^{l}x_{i}^{l-1}+b_{j}^{l})$$

where *l* is the network layer number, *k* denotes the convolution kernel, *m* denotes the size of the feature map’s receptive field on the initial image, and b denotes the offset.

Pooling layers are typically designed after convolutional layers to reduce feature dimensions, decrease parameters, and accelerate network training. This paper uses maximum pooling to process features. The expression of a feature map pooling can be expressed by Equation 2:

(2) $$x_{j}^{l}=f(\rho_{j}^{l}down(x_{j}^{l-1}+b_{j}^{i}))$$

where down() is the downsampling function, ρ and *b* represents different constants used when sampling feature maps.

Convolutional neural networks are typically equipped with fully connected layers in the last few layers to reduce the original high-dimensional feature vectors to low-dimensional feature vectors, remove redundant and noisy information from the high-dimensional feature vectors, and improve recognition accuracy.

### 2.2 Integrated convolutional block attention module

**Figure 2:**
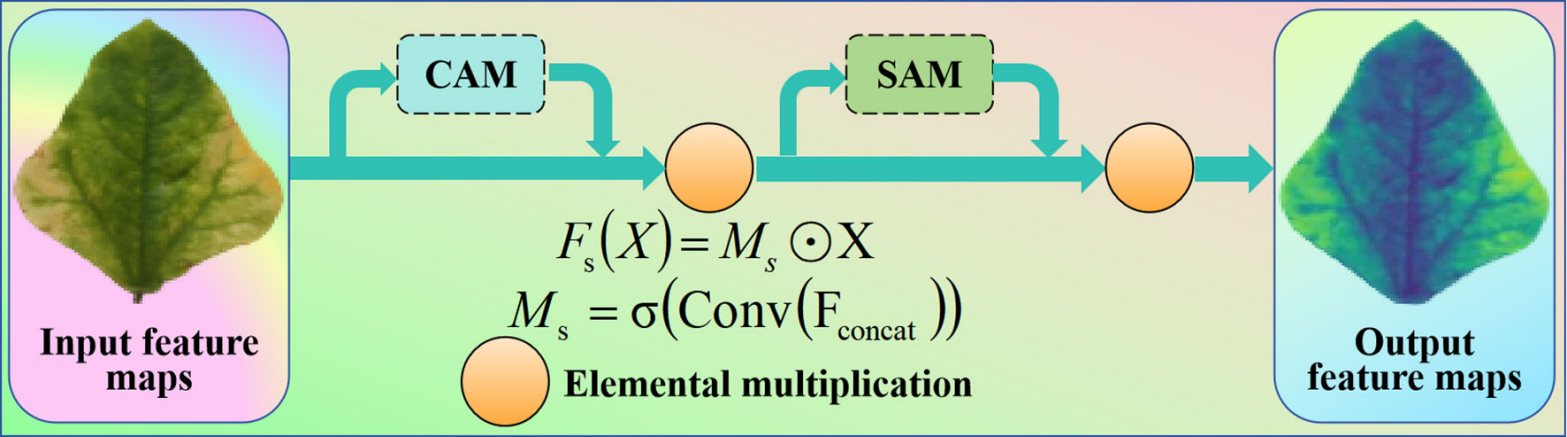
Proposed CBAM structure, effectively increase the characteristics of leaf disease.

To enhance feature discrimination in bean disease identification, we integrate the convolutional block attention module into the improved ZFNet. CBAM adaptively emphasizes informative channels and spatial regions through its dual attention mechanism, strengthening relevant features while suppressing redundant or noisy responses. This optimization improves feature representation capacity and model robustness against complex backgrounds, enhancing classification accuracy in horticultural image analysis. As illustrated in **Figure 2**, the module is inserted after specific convolutional layers, enabling efficient and interpretable feature refinement with minimal computational overhead.

Let the input feature map be X, its number of channels be C, and the global average pooling formula be (AvgPool). We operated on each channel c. The average AvgPool(X) of the C channels can be formulated by Equation 3:

(3) $$A\text{vg}P\text{ool}\left(X\right)\text{c}=\frac{1}{H\times W}\sum_{\text{i}-1}^{H}\sum_{\text{j}-1}^{W}X_{\begin{array}{l} \text{c},i,j \end{array}}$$

where *H* is height and *W* represents width.

Global maximum pooling (MaxPool) operates on each channel *c*, and the mathematical expression for the maximum value MaxPool(X) of C channels can be expressed by Equation 4:

(4) $$M\text{a}xPool\left(X\right)c,i^{'},j^{'}=\max_{i\in R,j\in R}X_{c,s\cdot i^{'}+i,s\cdot j^{'}+j}$$

The results of global average pooling and global maximum pooling are fed into a shared multilayer perceptron (MLP) or convolutional layer for feature transformation, respectively. Let the transformed features be FAvg and Res-unit. The MLP can be represented in the form of a linear transformation plus an activation function. It can be formulated by Equation 5:

(5) MLP(x)=σ(Wx+b)

where *W* is the weight matrix, *b* represents the bias term, and *σ* denotes the activation function.

The results of the feature transformations are summed, and the weight values, Mc, are generated by the Sigmoid function. It can be calculated by Equation 6:

(6) $$M_{\text{c}}=\text{σ(}F_{\text{Avg}}+F_{M\text{ax}})$$

The generated weight values are multiplied with each channel of the original feature map to achieve weighting of channel attention. It can be expressed by Equation 7:

(7) $${\text{F}}_{\text{c}}\left(X\right)=M_{\text{c}}⊙X$$

where ⊙ is the element-by-element multiplication.

Performs average pooling and maximum pooling on the input feature map X in the channel dimension to obtain two spatial feature maps: AvgPool(X) spatial and MaxPool(X) spatial. The results of average pooling and maximum pooling are spliced together to form a new feature map. The spliced feature maps are fused by a convolutional layer to generate the spatial attention map. They can be described by Equations 8, 9:

(8) $$F_{\text{concat}}=\text{concat}\left(A\text{vg}P\text{ool}{\left(X\right)}_{\text{spatial}},M\text{ax}Pool{\left(X\right)}_{\text{s}patial}\right)$$

(9) $$M_{\text{s}}=\text{σ}\left(\text{Conv}\left({\text{F}}_{\text{concat}}\right)\right)$$

where Conv is the convolution operation, and σ denotes the Sigmoid activation function.

Multiply the generated attention map with the original feature map X in the spatial dimension to achieve weighted spatial attention. It can be described by Equation 10:

(10) $$F_{\text{s}}\left(X\right)=M_{s}⊙X$$

The CBAM hybrid attention mechanism mainly combines channel attention and spatial attention to autonomously loop-learn features and make appropriate adjustments to channel and position parameters. Its mathematical expression is as Equation 11:

(11) F(X)=M⊙X

where *M* is the final attention map, and F(*X*) denotes the weighted feature map.

**Figure 3:**
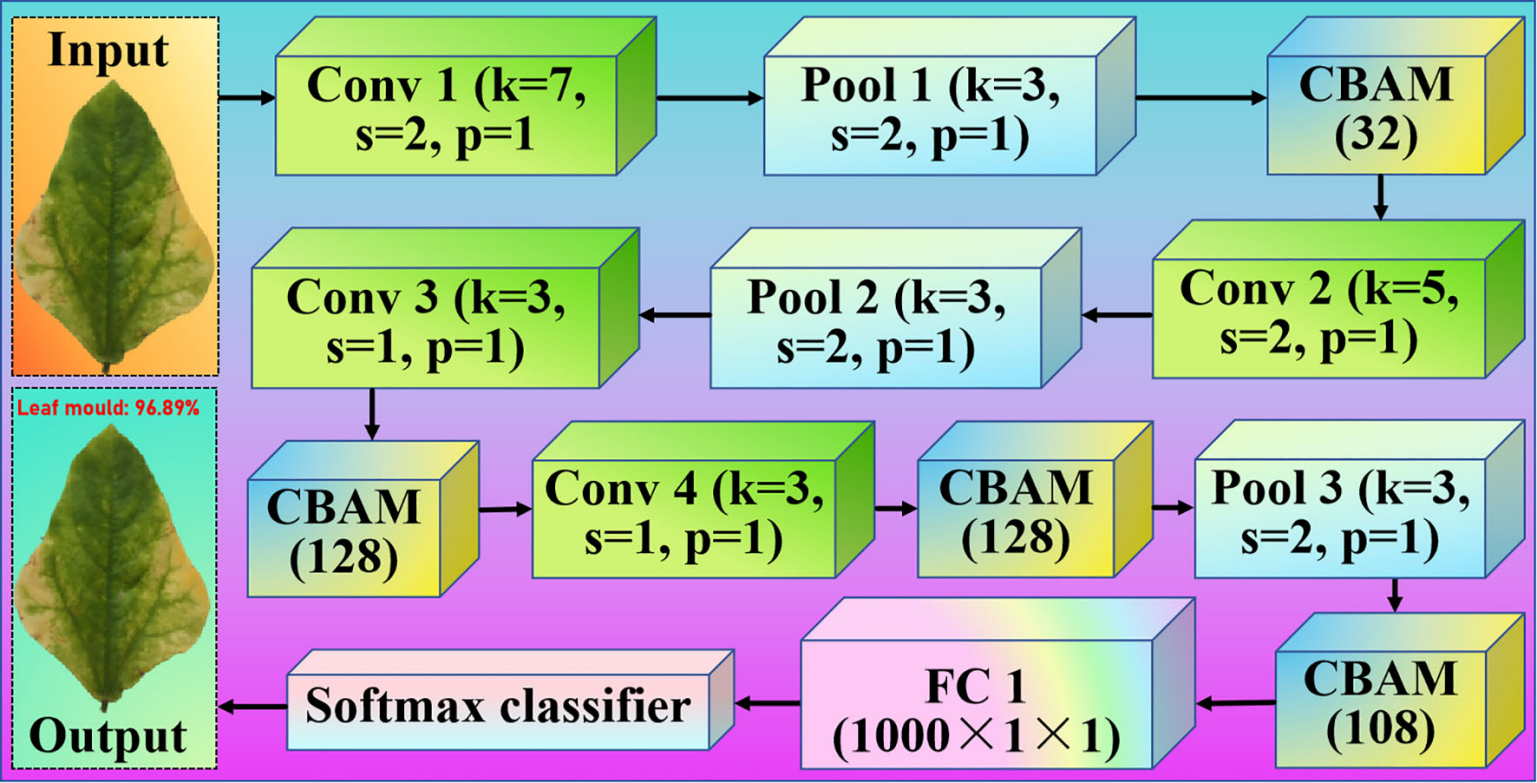
The improved ZFNet with the proposed CBAM.

We added the CBAM to the improved ZFNet. The overall network structure is sketched in **Figure 3**. We incorporated the CBAM into the enhanced ZFNet, which increased the model’s attention and discriminative capacity toward illness features while also improving recognition accuracy. However, it also increases computational complexity and may overfit on small datasets, leading to gradient vanishing. In the future, we plan to use lightweight attention variants, application data augmentation techniques, and transfer learning to reduce the number of computational parameters.

### 2.3 Fused Res-unit

Due to the proposed dual attention mechanism introduced earlier, the network training becomes unstable, which can easily lead to gradient vanishing and exploding. Therefore, we introduced the residual connection module, which connects the input directly to the output jump, so that the gradient can flow directly, alleviating the problem of gradient disappearance or explosion and enabling the network to be trained to become more stable. The residual unit (Res-unit) effectively tackles the degradation problem of deep networks by utilizing residual structures, allowing networks with complicated attention mechanisms to be reliably trained to deeper layers.

**Figure 4:**
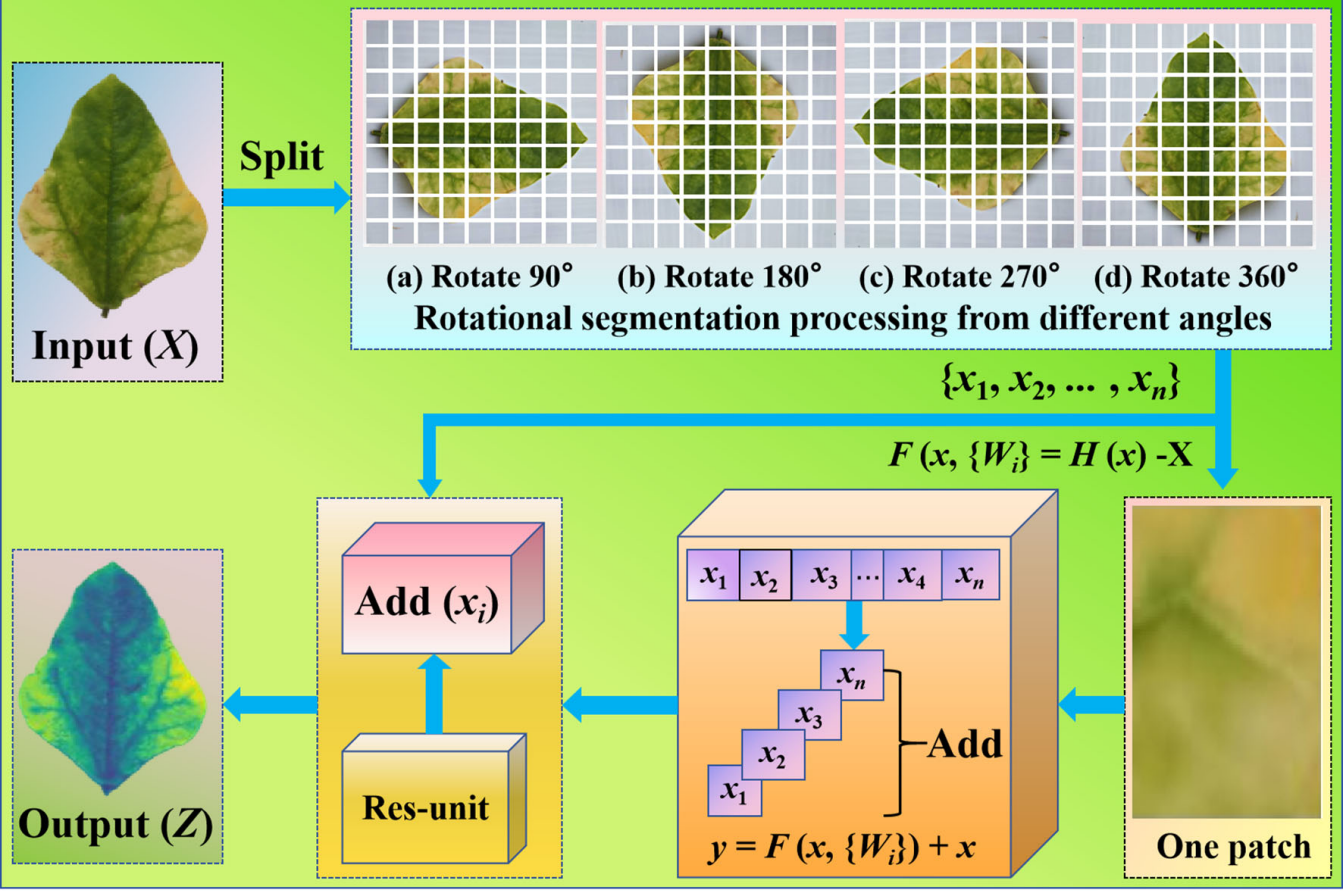
Proposed Res-unit structure for processing feature maps.

The most important aspect is that remaining connections function as feature protectors. It is transferred straight to the deep layer via identity mapping, which carefully screens and enhances the CBAM’s key feature information to prevent loss or dilution during future transformations. This not only greatly improves gradient flow, accelerates convergence, and enhances training stability, but also achieves effective fusion of the basic features before the CBAM optimization and the refined features after optimization. Ultimately, the CBAM’s dynamic focusing capability, ResNet’s nonlinear transformation capability, and residuals’ feature protection/transmission capability combine to dramatically overcome the model’s performance constraint and increase accuracy. The proposed Res-unit is elucidated in **Figure 4**.

Let the input be x, and the desired output be H(*x*), the Res-unit approximates the difference between H(*x*) and *x* by learning the residual mapping. It is as Equation 12:

(12) $$F(x,\left\{W_{i}\right\}=H(x)-X$$

where W is the weight parameter of the convolutional layer.

The final output *y* of the Res-unit can be expressed by Equation 13:

(13) $$y=F\left(x,\left\{W_{\text{i}}\right\}\right)+x$$

Where $F(x,\left\{W_{i}\right\})$ is the residual mapping learned through the main path convolutional layer, while *x* is the input passed directly through the jump connection.

**Figure 5:**
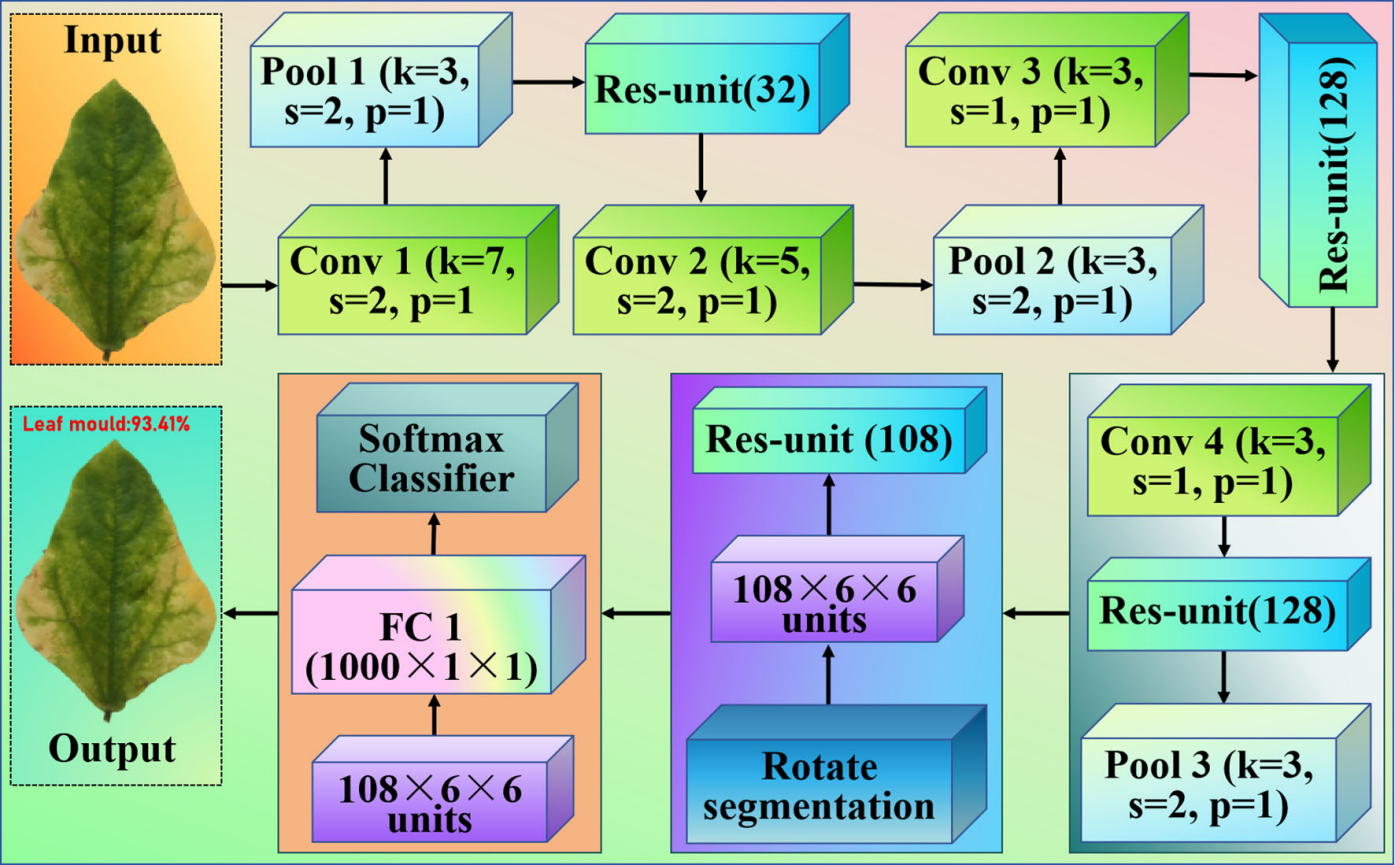
Improved ZFNet with the Res-unit.

We added the Res-unit to the improved ZFNet in **Figure 5**. We added a Res-unit to the improved ZFNet to enhance feature reuse, alleviate gradient vanishing, and improve the model’s deep expression ability. However, it raises the computational cost and the risk of overfitting, which must be reduced through regularization or lightweight design. Joining CBAM alone is superior to joining the Res-unit alone. This is because the lightweight attention mechanism CBAM can accurately focus on local features of diseases and has higher computational efficiency.

**Figure 6:**
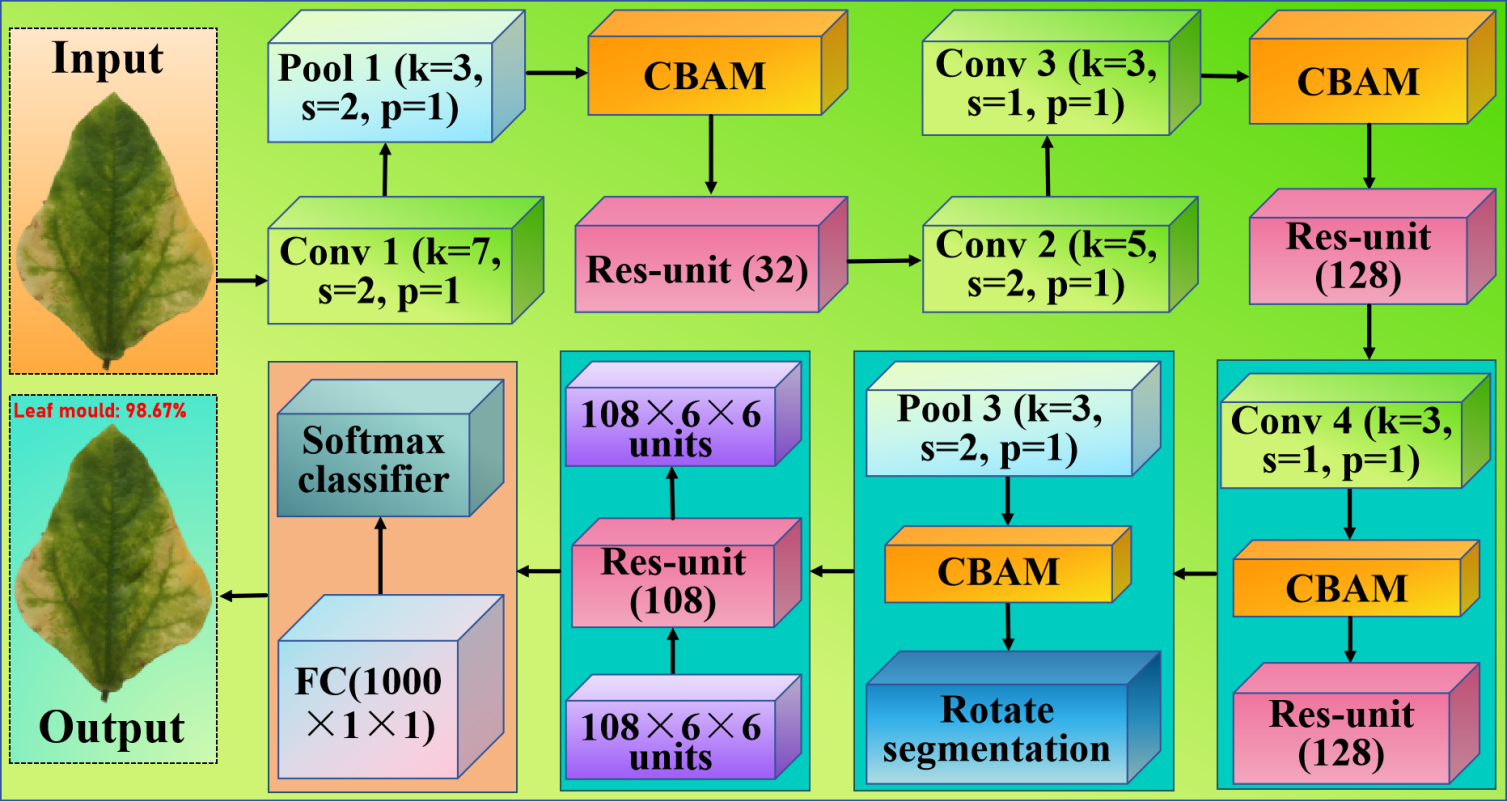
Proposed ZDAM model.

We added the CBAM and Res-unit to the improved ZFNet. The overall network structure is presented in **Figure 6**.

First, the classical ZFNet structure is simplified to improve feature extraction efficiency. Second, after simplifying the classical ZFNet pooling layer, the CBAM module is introduced to highlight the features of important locations in the bean leaf data through a dual attention mechanism of space and channel, thereby achieving comprehensive extraction of rich features in key areas. Finally, a residual module is added after each CBAM module, and a skip connection feature extraction method is introduced to avoid the vanishing gradient problem, enabling the network to learn the differences between input and output features more thoroughly, accelerate convergence, improve optimization performance, and enhance the model’s recognition performance.

## 3 Experimental results and analysis

### 3.1 Experimental equipment

The algorithm processing platform for the experiment is a personal computer equipped with an Intel Core RTX 4090 processor and an Intel Iris Xe Graphics Family graphics card. The software environment is Windows 11 x64, the programming language is Python 3.9, and the tool is PyCharm 2024.2.3. The deep learning framework PyTorch 2.4.1 is built on the server side to implement recognition training and testing.

### 3.2 Experimental data

**Figure 7:**
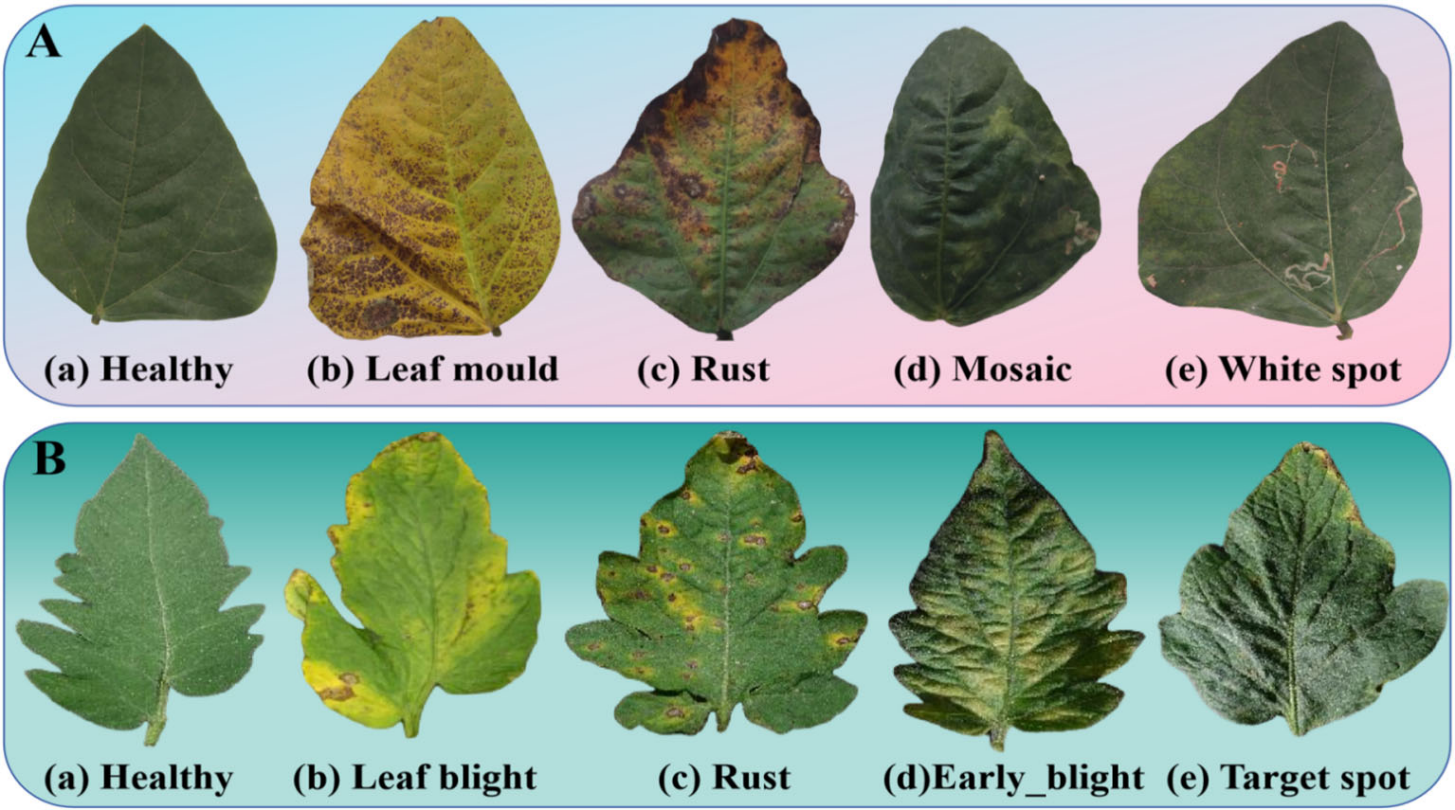
Samples of bean and tomato leaf diseases. **(A)** is our dataset. **(B)** is the public dataset.

The experimental data were partially sourced from a legume cultivation base in Henan Province, China, comprising a total of 11,903 samples. Additionally, to validate the model’s generalization capability for identifying fruit and vegetable leaf diseases, tomato leaf disease data from the open-source dataset New Plant Disease Dataset (https://www.kaggle.com/vipoooool/new-plant-diseases-dataset) were also utilized. Both datasets include healthy samples and four disease categories: rust disease, mosaic disease, leaf mold disease, and white spot disease. (https://pan.baidu.com/s/197Lyn2TGdIjLCE2gylsiHA?pwd=krpw). These images are in 24-bit color PNG format in **Figure 7**.

The dataset comprises 11,903 images: 2,976 raw captures and 8,927 images synthesized via the Pix2Pix generative adversarial network. As this project is in its early exploratory stage, the study faces limitations due to the seasonal and regional occurrence of bean leaf diseases in the field and uneven sample collection across disease types. Consequently, obtaining a sufficient number of real disease images with balanced categories is challenging in the short term. Therefore, we applied the Pix2Pix generative adversarial network to augment the limited real samples, enabling preliminary validation of the proposed ZDAM framework’s effectiveness and optimization potential during the early model development phase. All synthetic images were generated from original leaf samples. When splitting the training, validation, and test sets, we ensured that each subset contained both the original image and its corresponding synthetic image. Moreover, all synthetic images derived from the same original leaf were allocated entirely to the same subset alongside the original image. It includes healthy and four diseased tomato leaf categories (i.e., leaf mold, rust, mosaic, and white spot) in **Table 1**.

**Table 1:** The quantity of each category.

Categories	Healthy	Leaf mold	Rust	Mosaic	White spot	Total
Original data	646	637	670	585	438	2976
Augmented data	2138	1946	1856	1768	1219	8927
Total	2784	2583	2526	2353	1657	11903

We used a 3:1:1 data allocation strategy to distribute experimental data, which takes into account the characteristics and needs of the current research stage. The distribution of each category in the three subsets is presented in **Table 2**.

**Table 2:** The distribution of each type of data.

Categories	Train set	Val set	Test set	Total number
Healthy	1,610	536	537	2,683
Leaf mold	1,491	497	497	2,485
Rust	1,423	474	475	2,372
Mosaic	1,264	421	421	2,106
White spot	1,354	451	452	2,257
Total number	7,142	2,379	2,382	11,903

### 3.3 Train and test results

**Figure 8:**
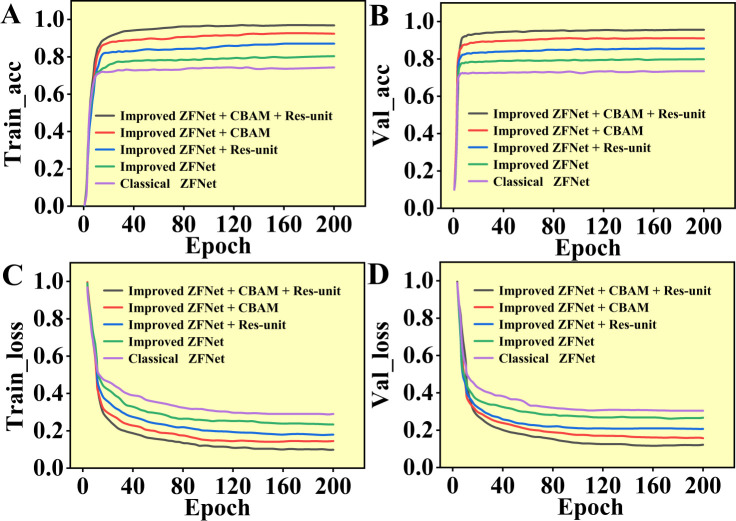
Accuracy and loss curves for different modules. The improved ZFNet model combined with CBAM and the Res-unit module performed best, with the training accuracy increased from 0.85 to 0.98–0.99, and the loss decreased from 0.21 to 0.1–0.15.

To compare and verify the feature extraction capabilities of the proposed model and the traditional ZFNet for bean disease data, we conducted iterative training and observed the changes trends in accuracy and loss values. The results are exhibited in **Figure 8**.

As shown in **Figure 8**, the proposed ZFNet with CBAM and residual units achieved the highest performance, with training accuracy stabilizing at 0.98–0.99 and loss decreasing to 0.10–0.15, outperforming all comparative models. In addition, training and validation curves indicate that adding only the CBAM module yields a higher accuracy increase rate and faster loss reduction than adding only the Res unit, along with a smoother convergence curve. CBAM leverages a dual-channel spatial attention mechanism to rapidly improve early training performance. In contrast, the Res unit offers more gradual gains. When combined, the model achieves the best performance with the fastest convergence and smoothest curve, demonstrating the complementary advantages of CBAM and Res unit in feature enhancement and gradient stability. The superior convergence stems from enhanced topology, CBAM’s channel-spatial refinement, and Resunit’s gradient stability, which deepen feature extraction for robust disease detection.

**Figure 9:**
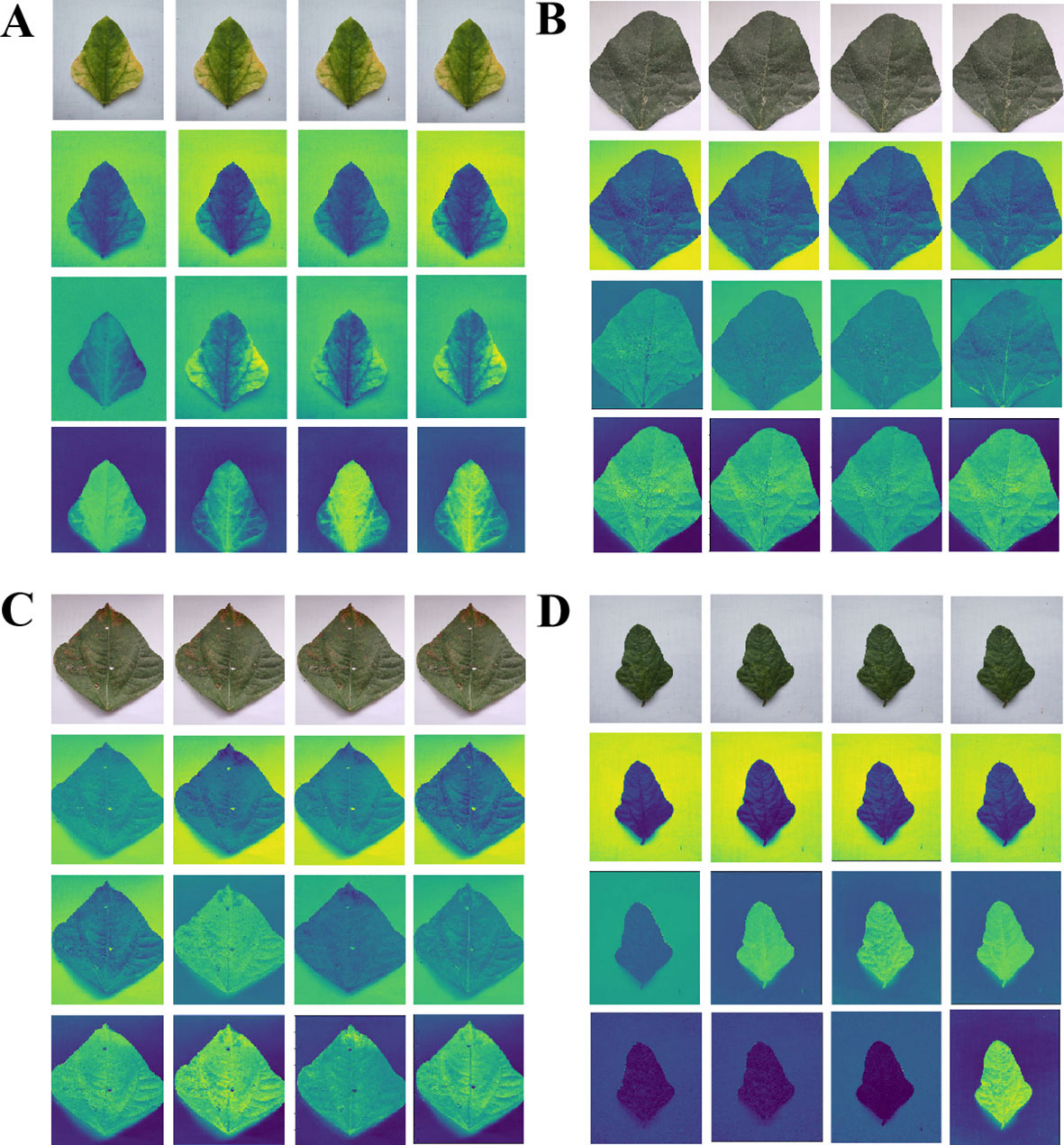
Features are extracted by convolution and pooling in turn, heat map of bean leaf diseases. **(A)** is leaf mold disease, **(B)** is mosaic disease, **(C)** is rust disease, and **(D)** is white spot disease.

To further validate the ability of the proposed ablation method to extract features of bean leaf disease, we used a heatmap to visualize the feature extraction of bean leaf disease in **Figure 9**.

**Figure 9** details the heatmap analysis. After convolution and pooling, the heatmap improves, concentrating responses along lesion edges, unlike the scattered activation after a single convolution. Specifically, adding only the Res-unit module yields a thermal response highly concentrated in the lesion area with precise edge localization and more complete preservation of small lesion features. In contrast, CBAM alone, while capable of focusing on discriminative regions, still exhibits some edge diffusion and insufficient activation intensity for certain small lesions, resulting in weaker deep feature transmission than the Res-unit. This demonstrates that the Improved ZFNet+CBAM+Res-unit module enhances lesion localization accuracy and feature representation synergy, contributing complementary gains without compromising classification performance. However, heatmaps provide only qualitative visualization; highlighted regions do not strictly represent the full basis for classification decisions due to information loss and approximation errors from gradient or activation computations, and they are sensitive to input perturbations and initialization methods.

**Figure 10:**
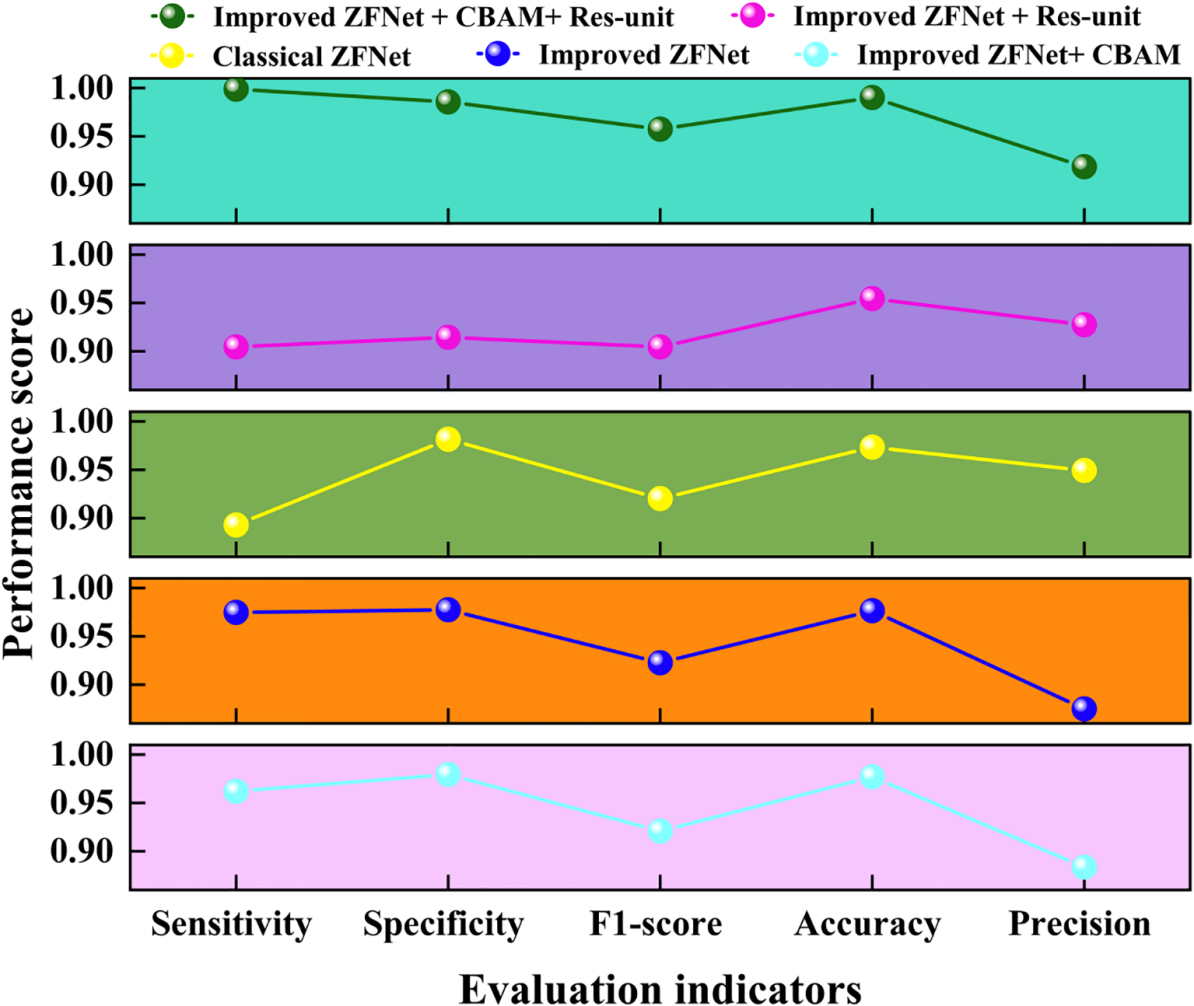
Comparison of evaluation indicators for different combinations. The improved ZFNet+residual unit+CBAM method is the best among the five evaluation indexes, and its F1-score is 2.77% higher than that of the improved ZFNet+CBAM method with the lowest performance.

To evaluate the proposed enhanced ZFNet with residual units and attention mechanisms, we conducted ablation studies using specificity, precision, sensitivity, F1-score, and accuracy. The results are presented in **Figure 10**.

As evidenced in **Figure 10**, the proposed ZFNet integrated with CBAM and ResNet achieves the highest scores across all five metrics. It outperforms the baseline ZFNet by 1.82–4.43% and the second-best variant (ZFNet+CBAM) by 0.45–1.56%. The attention mechanism prioritizes salient features and suppresses noise, improving adaptation to scale and deformation. Adding only CBAM yields slightly better gains than only Res-unit, highlighting the importance of channel-spatial attention for feature refinement, while the Res-unit enhances performance with minimal computational overhead. Specifically, adding either CBAM or Res-unit alone substantially improves various classification metrics compared to the original ZFNet. CBAM performs slightly better than ResNet, as it effectively focuses on lesion discriminative features through channel and spatial attention mechanisms while suppressing background interference. ResNet also delivers stable performance gains, with its skip connections offering unique advantages in deep feature transfer. Given their distinct strengths, CBAM and Res unit complement each other, and their combined use helps balance accuracy and stability. These results validate the integrated design for accurate bean disease identification.

To further validate the inference speed, robustness, and feasibility of edge deployment of the proposed model, we tested the parameters (Params), floating-point operations (FLOPs), and running speed of five models in an environment with 16 g of memory and an Intel Core i5-13500H graphics card. The specific results are depicted in **Table 3**.

**Table 3:** Comparisons of resource consumption parameters for different models.

Ablation models	Params (M)	FLOPs (G)	Speed (ms)	Accuracy(%)
Classical ZFNet	21.60	1.0	10	95.46%
Improved ZFNet	15.40 (↓6.20)	0.75 (↓0.25)	9.60 (↓0.40)	97.35% (↑1.89%)
Improved ZFNet+ CBAM	16.80 (↓4.80)	1.29 (↑0.29)	9.22 (↓0.78)	97.70% (↑2.24%)
Improved ZFNet + Res-unit	24.87 (↑3.27)	1.57 (↑0.57)	9.35 (↓0.65)	97.67% (↑2.21%)
Improved ZFNet + CBAM + Res-unit	26.66 (↑5.06)	1.64 (↑0.64)	9.25 (↓0.75)	99.02% (↑3.56%)

As can be seen in **Table 3**, the optimized ZFNet reduces the number of parameters, FLOPs, and latency and increases accuracy. After adding the CBAM to the simplified ZFNet, both the number of parameters and latency were reduced. The delay in joining the Res-unit has slightly increased, and compared to joining the CBAM, the FLOP has increased by 0.13. This indicates that CBAM performs slightly better than the Res unit. Adding either the CBAM or Res unit alone improves performance over the original ZFNet. CBAM offers clear advantages in parameter count, FLOPs, and inference speed, achieving an accuracy of 97.70%, slightly higher than the 97.67% of Res unit. This indicates that the CBAM uses computational resources more efficiently while delivering marginally better classification performance, making it more suitable as a core module for lightweight improvement. Although Res unit incurs higher parameter count and computational overhead, its accuracy still reaches 97.67%, a 2.21% improvement over the original ZFNet. This demonstrates that its skip connections play an irreplaceable role in stable deep training and feature transfer. When we add the CBAM and Res-unit simultaneously, the number of model parameters is higher than in other combined models. It achieves the optimal balance between the number of parameters and speed. Overall, compared with the classical ZFNet, the proposed model has increased the number of parameters by 5.06M and FLOPs by 0.64G. This indicates that the model effectively increases the number of computational parameters, reduces latency, and is more conducive to lightweight edge deployment while balancing parameter quantity, FLOP, and inference speed.

**Figure 11:**
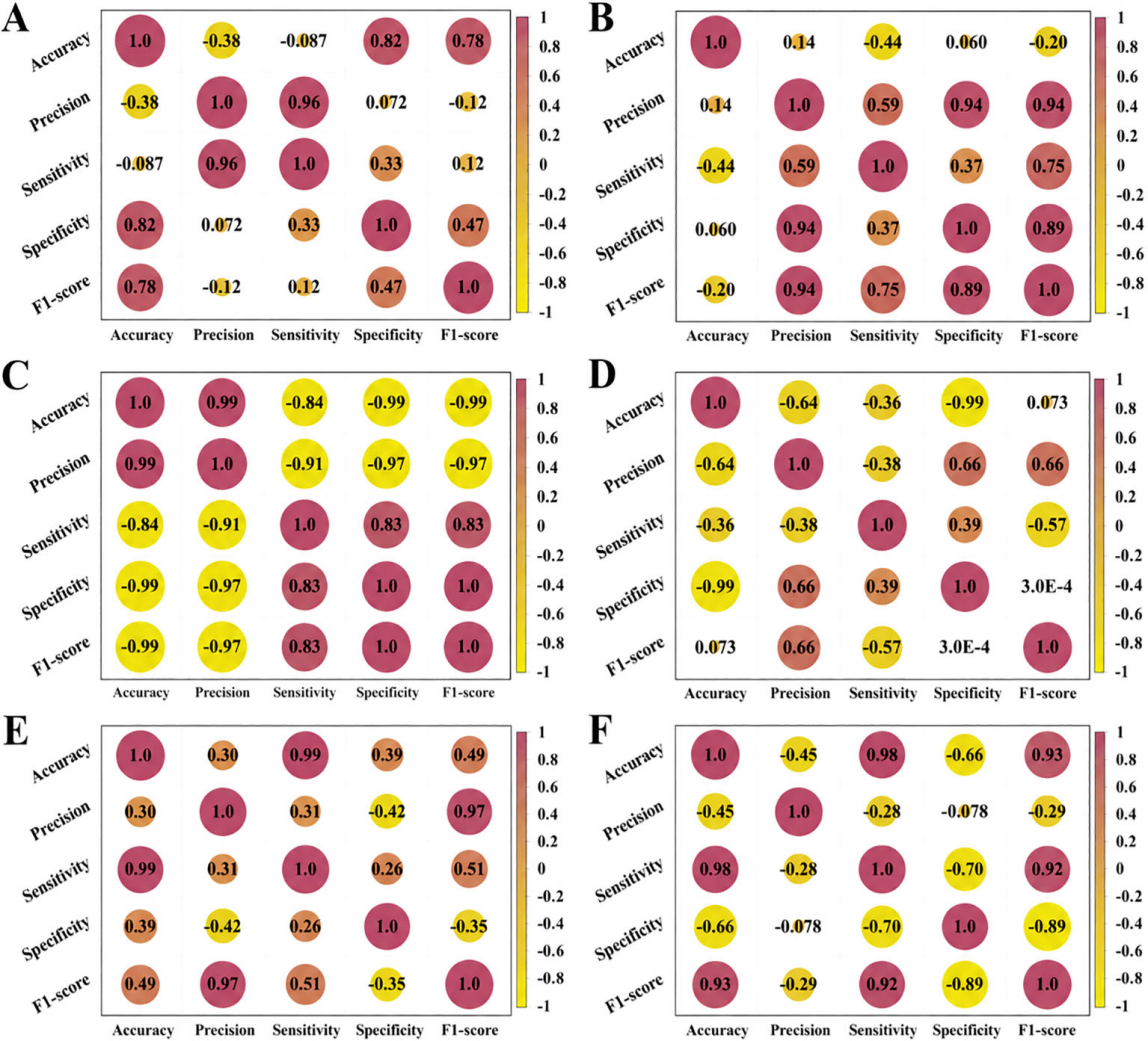
Heat maps of the correlation between evaluation indicators of different models. **(A–F)** are the correlation heatmap analysis results of the Classical ZFNet, Classical ZFNet+CBAM+Res-unit, Improved ZFNet, Improved ZFNet+CBAM, Improved ZFNet+Res-unit, and Improved ZFNet+CBAM+Res-unit, respectively.

A correlation heatmap visually encodes the strength of Pearson correlation coefficients between variables through color intensity. Each matrix cell represents a coefficient ranging from -1 (perfect negative correlation) to 1 (perfect positive correlation), with 0 indicating no linear relationship. We employed this method to analyze interrelationships among performance metrics across different models and assess their collaborative capabilities in multidimensional evaluation. The resulting correlation pattern is presented in **Figure 11**. According to **Figure 11**, in the classical ZFNet, accuracy was negatively correlated with precision (r = −0.38), whereas sensitivity was positively correlated with the F1-score (r = 0.12). The improved ZFNet with CBAM and residual units showed a strong positive correlation between specificity and F1-score (r = 0.89). The improved ZFNet with residual units achieved an almost perfect positive correlation between accuracy and sensitivity (r = 0.99). In contrast, the skip connections in the Res unit yield an almost perfect positive correlation between accuracy and sensitivity (r = 0.99). This indicates that the model can enhance overall classification accuracy while simultaneously improving recall, with highly coordinated metrics that avoid performance tradeoffs caused by weak or negative correlations. Although CBAM alone also shows a positive correlation between accuracy and specificity (r = 0.66), its degree of synergy is significantly lower than that of the Res unit and lacks the same strong consistency. Therefore, the ZFNet with Res unit model is superior to the ZFNet with CBAM in balancing these metrics. However, the correlation heatmap reflects only linear dependence among evaluation metrics and cannot reveal specific performance fluctuations on individual samples or challenging cases. The positive or negative correlations shown may mask nonlinear relationships or conditional dependencies between metrics. Furthermore, the interpretation of the heatmap heavily depends on the sample distribution and the set composition. Different partitioning methods can significantly alter the correlation structure. Overall, the proposed model has better specificity and robustness while maintaining high accuracy.

**Figure 12:**
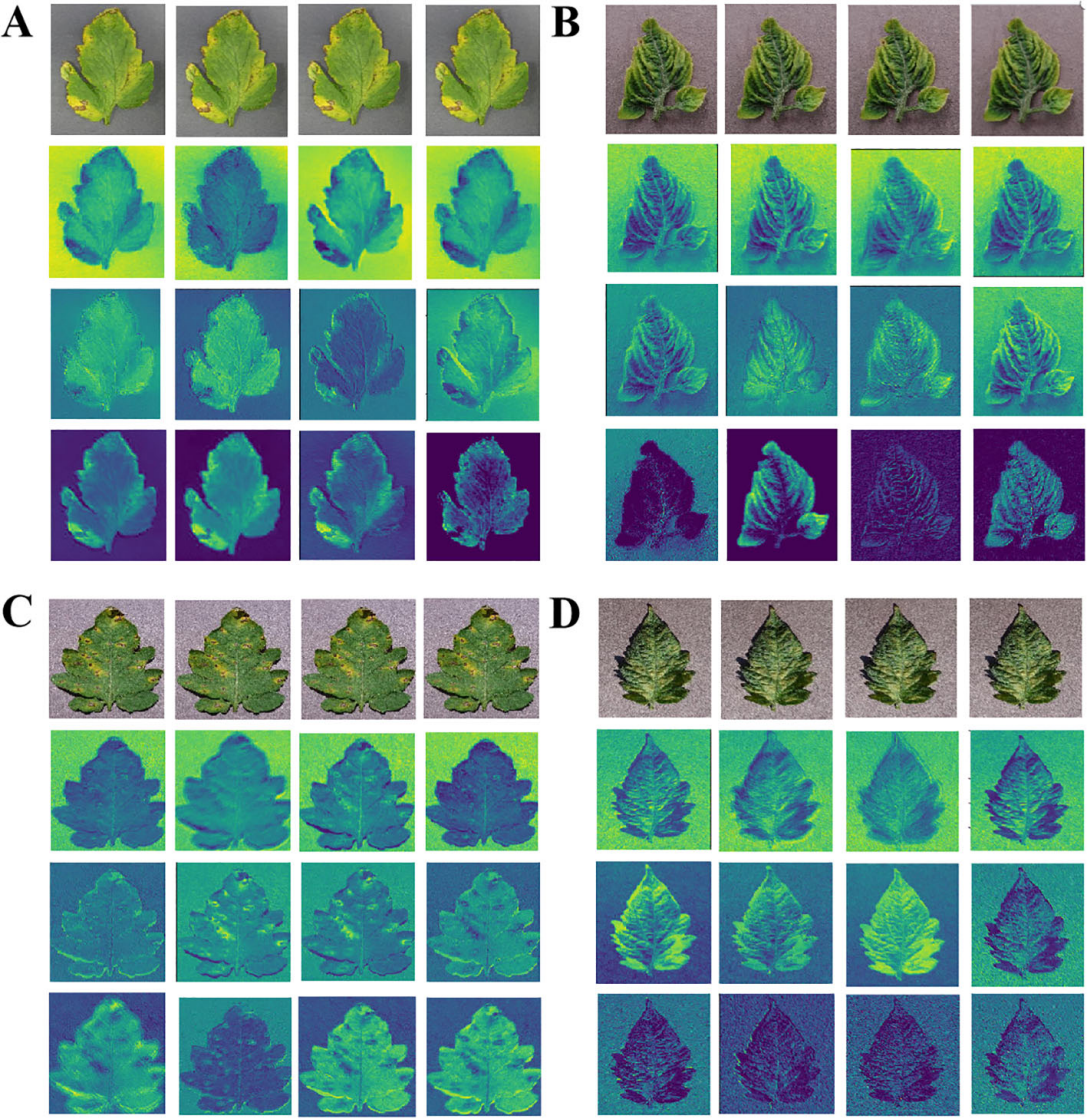
Features are extracted by convolution and pooling in turn, heat map of tomato leaf diseases. **(A)** is leaf mold disease, **(B)** is mosaic disease, **(C)** is rust disease, and **(D)** is white spot disease.

To validate the accuracy of the model’s feature extraction analysis, this paper also performed feature extraction on tomato leaves and analyzed whether the heatmap results accurately identified the characteristics of the diseased areas. The results are explained in **Figure 12**.

The comparison verification in **Figure 12** shows that the single-layer convolutional heatmap has issues such as scattered response, poor capture of tiny lesions, and coarse edge localization. Specifically, in the heatmap for tomato leaf diseases, adding only Res unit produces a response more concentrated in the lesion area, with better edge localization and more complete preservation of deep lesion features than adding only CBAM. Although CBAM alone can focus on discriminative regions, it insufficiently suppresses background noise against complex tomato leaf backgrounds, leaving residual responses in some disease-free areas. The improved ZFNet with CBAM and Res unit produces a heatmap showing lesion edge response, spatial information, and small lesion capture, confirming improvement in localization and feature complementarity without performance loss. However, heatmap analysis for tomato leaves inherits the limitations of qualitative visualization. Heatmaps display only relative activation intensity from convolutional layers, failing to accurately reflect model confidence differences between lesion and healthy areas or to distinguish correct from accidental activation. Under uneven lighting, leaf overlap, or complex backgrounds, high response regions may be dominated by noise or artifacts, while true small lesions may respond inadequately. Thus, these results should serve only as an intuitive auxiliary reference, not as decisive evidence of model robustness.

We used ablation experiments to verify the contribution of each newly introduced strategy to the classification capabilities of different models. The comparison results are presented in **Table 4**.

**Table 4:** Comparison of evaluation indicators for ablation experiments on an open-source dataset.

Ablation models	Recall	Precision	Specificity	F1-score
Classical ZFNet	95.25%	96.22%	96.41%	95.73%
Improved ZFNet	97.65% (↑2.40%)	97.46% (↑1.24%)	97.24% (↑0.83%)	99.22% (↑3.49%)
Improved ZFNet+ CBAM	98.21% (↑2.96%)	98.56% (↑2.34%)	98.08% (↑1.67%)	98.32% (↑2.59%)
Improved ZFNet + Res-unit	98.78% (↑3.53%)	99.01% (↑2.79%)	98.48% (↑2.07%)	98.89% (↑3.16%)
Improved ZFNet + CBAM + Res-unit	99.03% (↑3.78%)	99.30% (↑3.08%)	99.56% (↑3.15%)	99.16% (↑3.43%)

As shown in **Table 4**, the improved ZFNet achieved faster speed and a 1.16% higher F1-score than the classic ZFNet. Adding Res unit raised precision by 2.34%; adding CBAM increased specificity by 2.07%, enhancing feature representation and complex background adaptation. In contrast, on this open-source dataset, the recall, accuracy, specificity, and F1-score of the Res unit alone exceed those of the CBAM alone. ResNet transfers deep semantic features through skip connections, fully leveraging its feature extraction advantage for the complex disease morphology in this dataset. Although the CBAM can focus on discriminative regions, its ability to preserve deep features when used alone is relatively limited. When the CBAM and Res-unit modules are integrated synergistically, recall increases by 1.38% and 3.78% compared to the improved ZFNet and classic ZFNet, respectively. This suggests that the two modules have good structural complementarity, effectively enhancing the model’s sensitivity and robustness to bean leaf disease spots while ensuring lightweighting.

**Figure 13:**
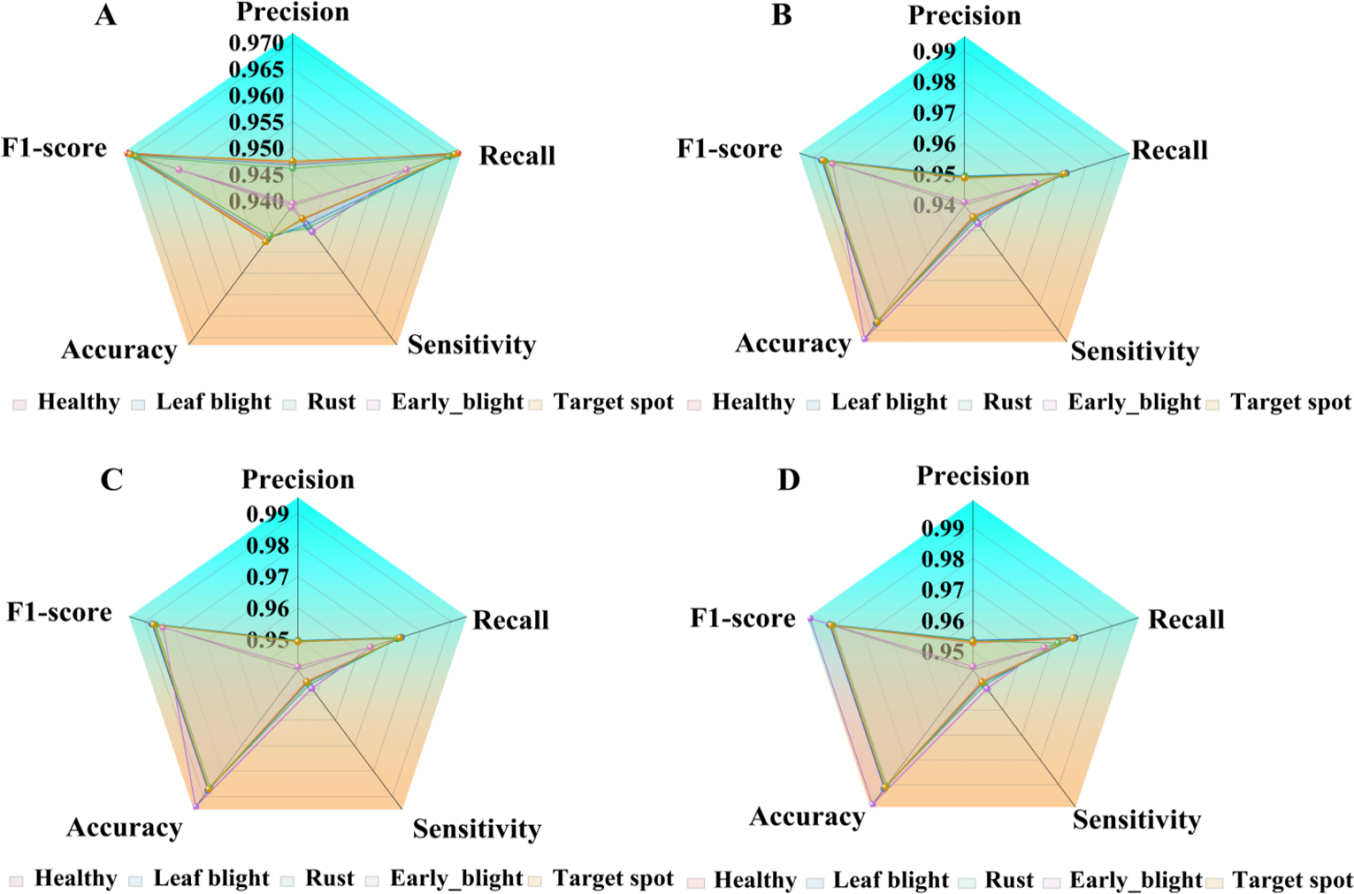
Radar chart for different disease types. **(A–D)** are the radar chart results of the improved ZFNet, Improved ZFNet+CBAM, Improved ZFNet+Res-unit, and Improved ZFNet+CBAM+Res-unit, respectively.

A radar chart is an efficient visual aid for demonstrating the relative benefits and drawbacks of various models in a variety of leaf disease classification tasks because it can graphically show several performance metrics in a single coordinate system. This visualization technique helps to highlight the model’s potential weaknesses for specific types of leaf diseases. To systematically analyze the variances in diagnostic capacities of various models, we employed radar charts to compare classification performance. The results are presented in **Figure 13**.

As can be seen in **Figure 13**, the proposed model surpasses the three comparative models across all metrics (recall, precision, sensitivity, F1-score, accuracy). Both the improved ZFNet+CBAM and ZFNet+Res-unit variants outperform the traditional ZFNet, with the latter showing a slight edge over the former. Our approach shows more obvious advantages for the leaf mold and flower leaf categories, reflecting the strong adaptability of Res-unit skip connections in transmitting deep features for bean diseases. Meanwhile, the indicators for CBAM alone remained above 95%, particularly for white spots and rust, where the gap compared to the Res unit was small. This indicates that the attention mechanism remains effective at focusing on diseased areas. The proposed model achieves consistent performance, exceeding 95% for all four disease categories, with each metric above 97%. The recognition probability for leaf mold ranges between 0.65–0.70, while white spot exhibits a marginally higher distribution. Overall, the model demonstrates stable and highly accurate identification of four bean leaf health states, supporting its practical value for objective, multi-disease early-warning systems in real-world crop production.

**Figure 14:**
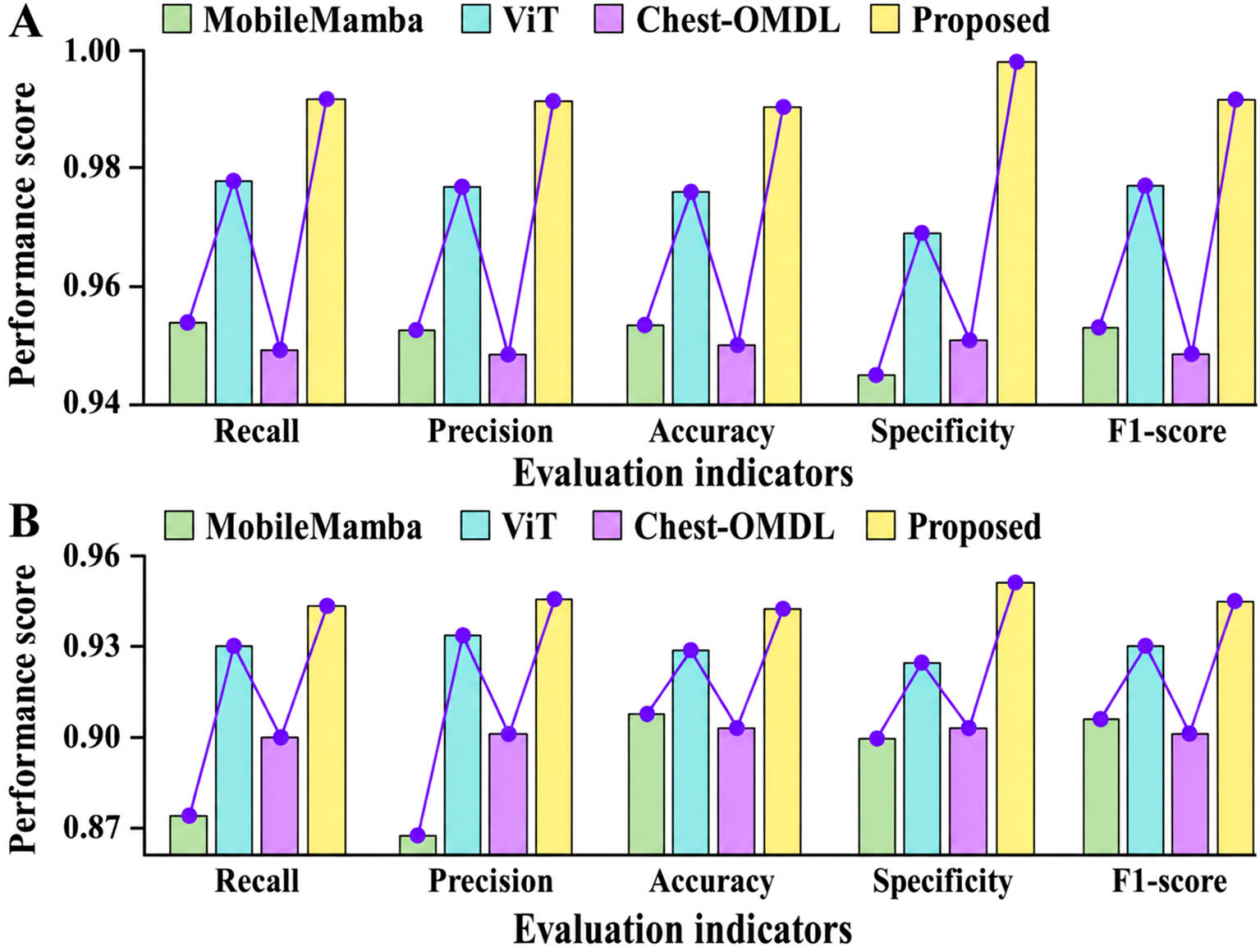
Comparison of evaluation indicators for different models. **(A)** is tested using bean data. **(B)** is tested using open-source tomato data.

To evaluate the proposed method, we compared it with MobileMamba, Vision Transformer, and Chest OMDL on a self-collected green bean dataset and a public tomato dataset using recall, precision, sensitivity, F1-score, and accuracy. The results are shown in **Figure 14**.

As illustrated in **Figure 14**, our ZDAM exhibits a comprehensive advantage over other deep learning models across five metrics on both the bean and public tomato datasets. Compared to Chest-OMDL, it enhances feature extraction despite using fewer layers. Versus MobileMamba, it achieves higher accuracy with a more efficient architecture. Although Vision Transformer is efficient, our model attains superior accuracy with significantly less training time. These improvements stem from the dual-attention mechanism, which refines feature extraction, and the residual partial convolutions, which reduce redundancy, thereby lowering parameters and boosting computational efficiency.

**Figure 15:**
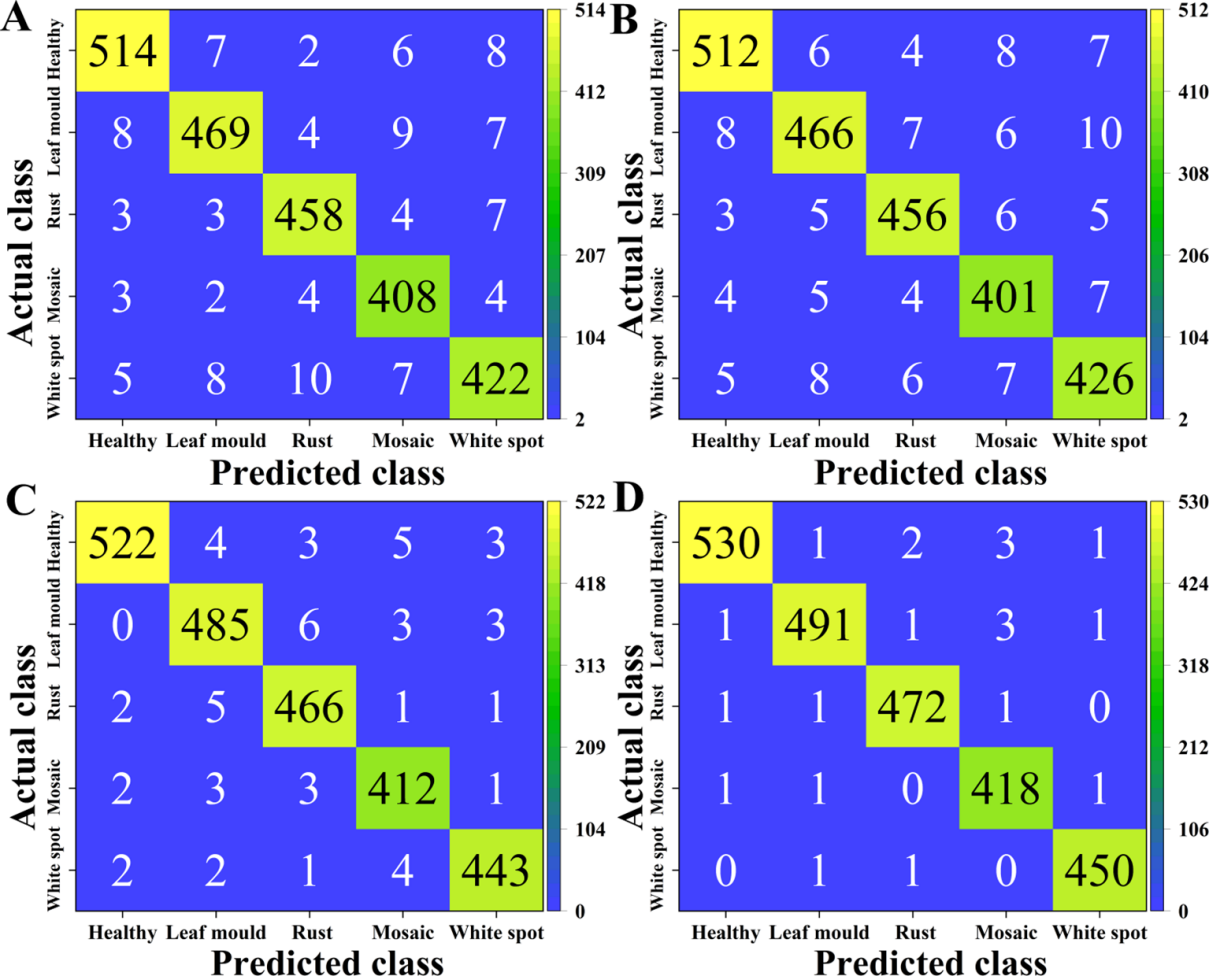
The classification results of confusion matrices. **(A–D)** are MobileMamba, Chest-OMD, Vision Transformer, and the proposed approach, respectively.

We used the confusion matrix in **Figure 15** to compare classification results for bean diseases across models. Diagonal entries represent correctly classified samples, while off-diagonal entries show misclassifications. This reveals which categories are well distinguished and which are easily confused.

As shown in **Figure 15**, the proposed model achieved the highest overall classification accuracy (99.2%), outperforming Vision Transformer (97.61%), Chest-OMDL (95%), and MobileMamba (95.34%). Its performance was marginally lower for Mosaic recognition. This challenge arises from the disease**’**s complex lesion morphology, extensive symptomatic areas, and color similarity to other conditions, such as Leaf Mold, which collectively complicate feature differentiation. Nonetheless, the proposed method demonstrates high overall accuracy for bean disease identification.

**Figure 16:**
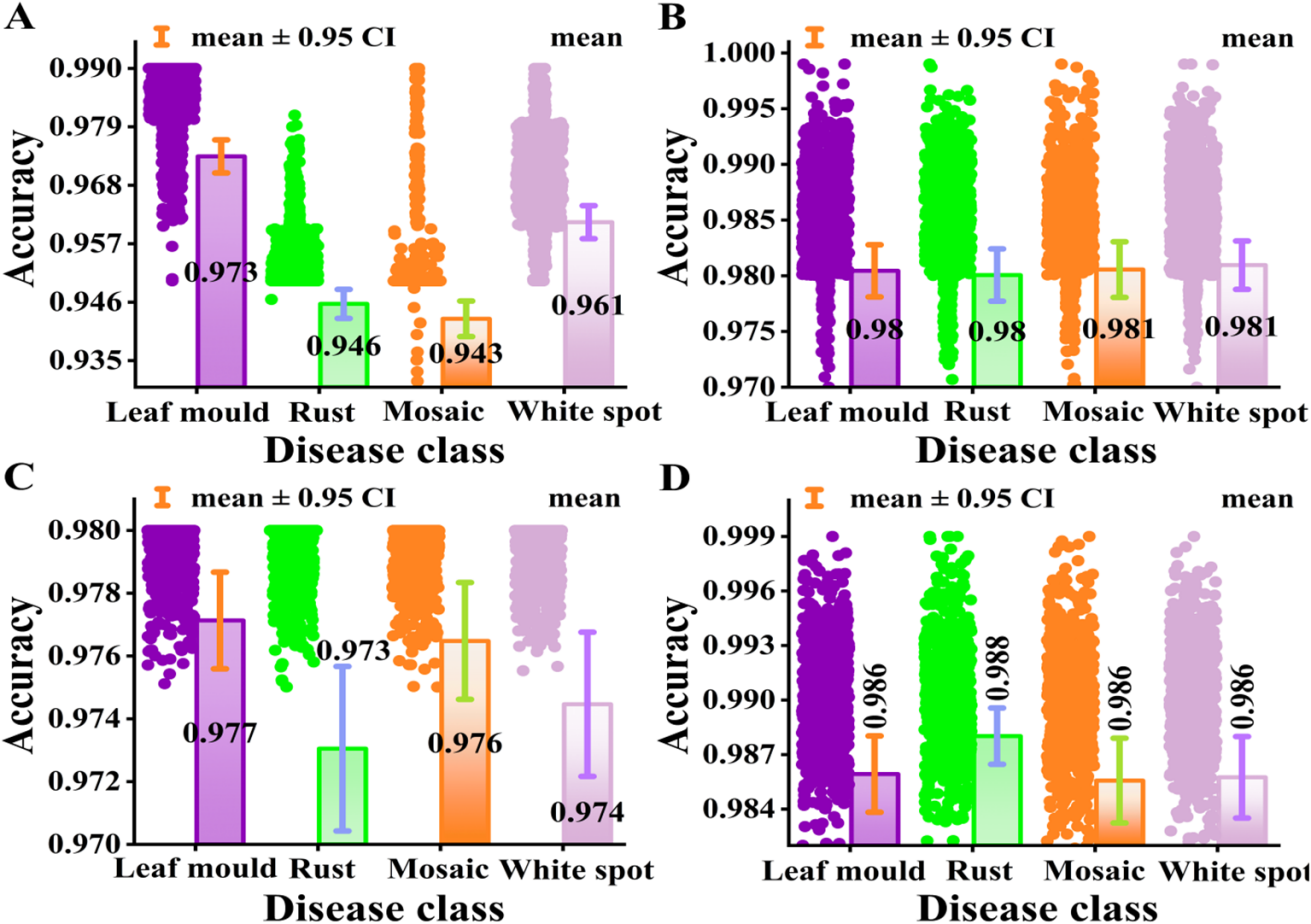
Mean error test results of four methods. **(A)** is MobileMamba, **(B)** is Chest-OMDL, **(C)** is Vision Transformer, and **(D)** is the proposed method.

**Figure 16** presents interval plots with error bars to display the 95% confidence interval for the mean recognition accuracy per disease category, showing the data fluctuation range and estimating the average accuracy. This allows comparison of the proposed method**’**s probability confidence with other methods and significance testing on the mean errors of each model.

As revealed in **Figure 16**, the proposed method achieved the highest mean accuracy in detecting four leaf diseases, surpassing the Chest-OMDL baseline. While MobileMamba demonstrated enhanced overall recognition and a tighter confidence interval upper bound, and the Vision Transformer exhibited greater stability with a reduced interval, our integrated approach delivered superior performance across all diseased categories. Error bar analysis confirms that the proposed scheme not only improves accuracy but also enhances result stability.

To verify the generality, class imbalance, and robustness of the proposed method for identifying different plant leaf diseases, we tested it using different open-source plant leaf disease datasets. This result is revealed in **Table 5**.

**Table 5:** Comparison of test results for different plant leaf diseases.

Dataset names	Recall	Precision	F1-score	Specificity
Our data	99.03%	99.30%	99.16%	99.56%
PlantVillage	98.80% (↓0.23%)	99.40% (↑0.10%)	99.09% (↓0.07%)	99.70% (↑0.14%)
CVPR 2020-fgvg7	98.88% (↓0.15%)	99.50% (↑0.20%)	99.18% (↑0.02%)	99.52% (↓0.04%)
New plant disease dataset	99.01% (↓0.02%)	99.20% (↓0.10%)	99.10% (↓0.06%)	99.50% (↓0.06%)

**Table 5** summarizes the proposed model’s performance on three public plant disease datasets. It closely matches comparative methods in specificity and F1-score. Our model exceeds the PlantVillage baseline by 0.10% in precision and outperforms the CVPR 2020-fgvg7 data by 0.15% in recall. While minor fluctuations occur relative to our proprietary bean dataset, the results confirm the method’s robust generalizability across diverse plant leaf disease identification tasks.

**Figure 17:**
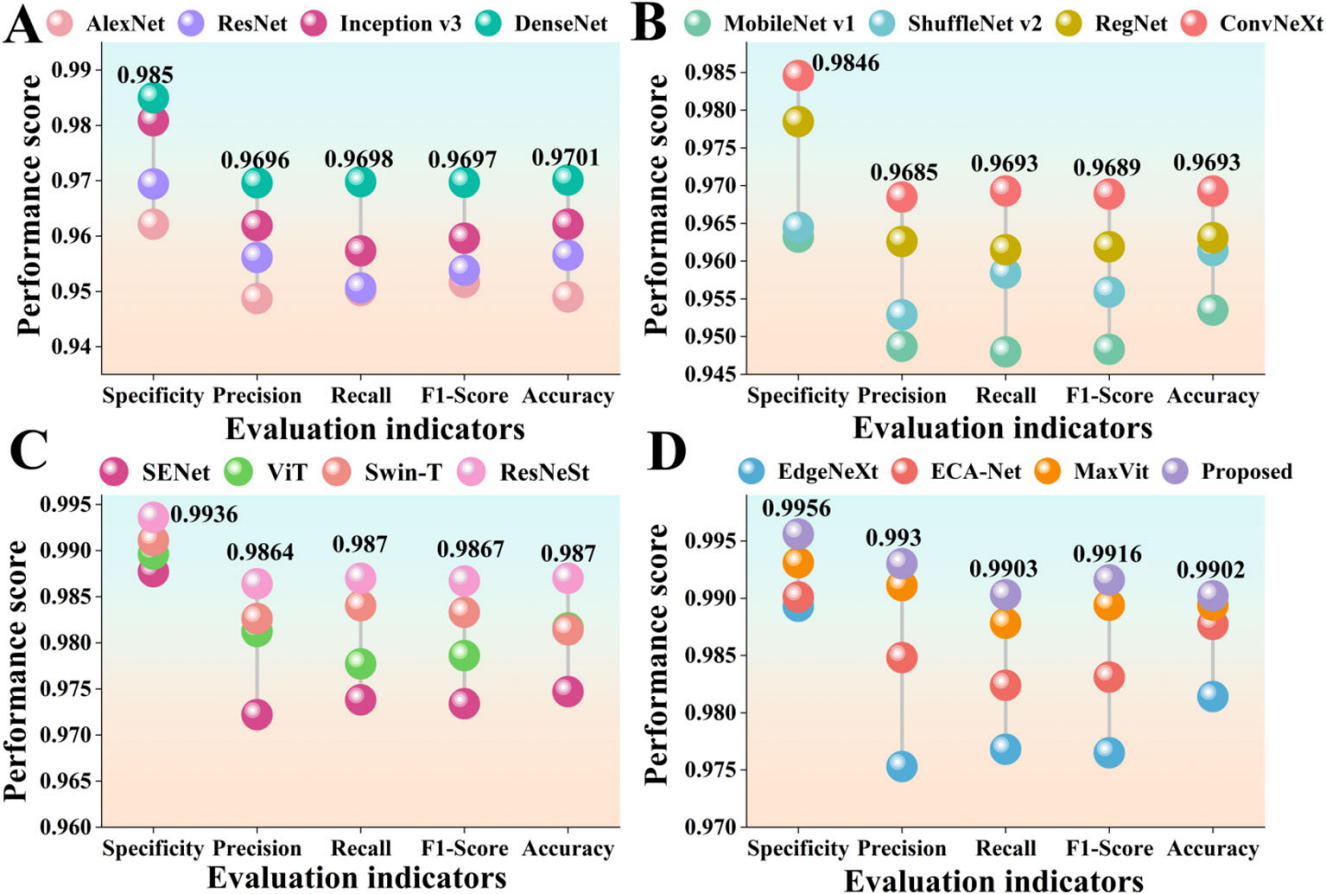
Performance indicators of different models. **(A)** is the classical deep learning models. **(B)** is Lightweight deep learning models. **(C)** is a deep learning models with attention mechanisms. **(D)** is the latest deep learning model.

To more intuitively compare the performance of different deep learning models on plant disease classification, we used a lollipop chart, and the corresponding test results are presented in **Figure 17**.

**Figure 17** reports that the proposed model outperforms other deep learning models across all evaluation metrics, verifying the collaborative enhancement between modules, and confirming the effectiveness of this method. For example, its accuracy is 2.01% higher than EfficientNet, sensitivity 1.1% higher than ConvNext, and accuracy 2.34% higher than DenseNet. Overall, the model achieves balanced performance across multiple indicators and demonstrates superior classification ability.

**Figure 18:**
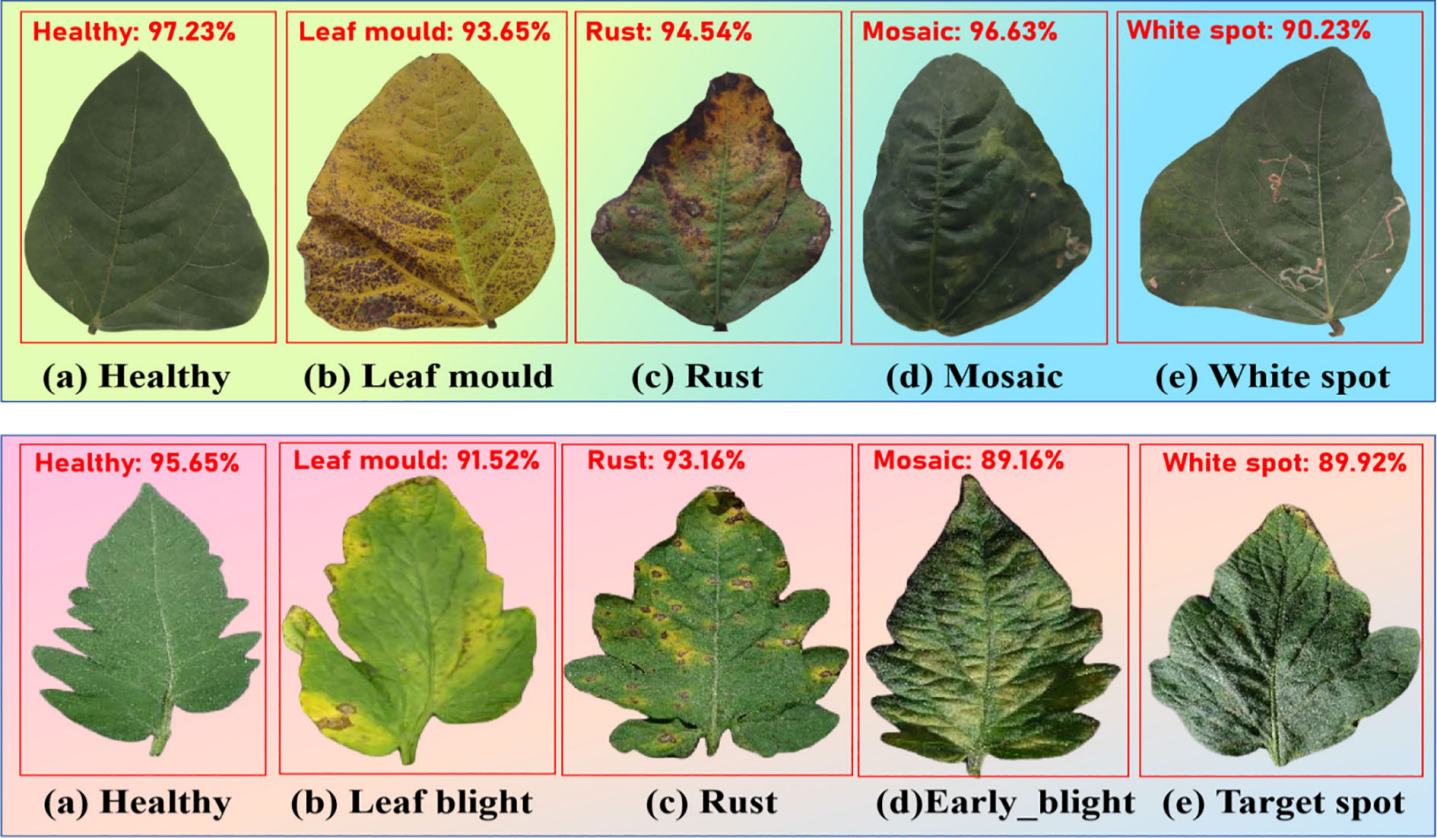
Prediction results. **(A)** is our data. **(B)** is the open-source data. **(a)** is healthy conditions, **(b)** represents leaf mold disease, **(c)** means rust disease, **(d)** is mosaic disease, and **(e)** is white spot disease.

We used the proposed model to randomly select some samples for testing, and the results are described in **Figure 18**. All predictions were correct, and the classification accuracy rate was above 89%.

## 4 Discussion

In recent years, deep learning has significantly advanced the field of plant leaf disease classification. For instance, Khan et al. (2024) explored plant disease image classification using a dense Inception architecture integrated with an attention module. To address challenges such as similar visual appearance, large intraclass variation, and changes in scale and resolution across different plant diseases, they proposed a hybrid network combining DenseNet and InceptionNet. An attention module was introduced to extract the most distinctive features while suppressing irrelevant information. The effectiveness of this method was validated on publicly available challenging datasets. Additionally, Raza et al. (2025) conducted a benchmark study of YOLO models for crop growth and weed detection in cotton fields, systematically evaluating 19 YOLO variants from YOLOv3 to YOLOv11 on the Cotton 8 dataset. Their results showed that larger models achieved the best detection accuracy (mAP@0.5 = 81.5%), whereas lightweight models offered the fastest inference speed with slightly lower accuracy. More importantly, the study identified that the key bottleneck lies in small object detection and precise localization rather than in network architecture design, and that detecting broadleaf and gramineous weeds remains challenging. Nevertheless, the above studies have certain limitations. The former relies primarily on image data from controlled environments, with limited validation of robustness under field conditions involving complex backgrounds, lighting changes, occlusion, and small disease spots. The latter, although focused on real farmland scenarios and systematically analyzing the tradeoff between speed and accuracy, is tailored to object detection tasks in cotton weed scenarios. Hence, the direct applicability of its conclusions to the localization of small lesions and interclass similarity issues in bean leaf disease classification still requires further verification.

The classification of bean leaf diseases faces several challenges, including complex background interference in the field, varying lighting conditions, difficulty in detecting small disease spots, leaf occlusion, and visual similarity among different disease categories (e.g., leaf mold and rust, mosaic and vitiligo). To address these issues, this study proposes the ZDAM model, which is based on an improved ZFNet and integrates a dual attention mechanism with residual units. Through structural simplification, embedding of CBAM, and residual connections, the model achieves an average recognition accuracy of 99.02% with only 9.32 MB of parameters, outperforming mainstream models such as MobileMamba and Vision Transformer. This demonstrates the effectiveness of the proposed framework in resource-constrained scenarios.

Although the ZDAM model achieves excellent classification performance on standard datasets, its limitations must be acknowledged. First, while the experimental data include some natural backgrounds, they do not systematically collect or quantitatively evaluate data under extreme conditions such as complex field backgrounds, varying light intensities (e.g., strong light, shadows, backlighting), leaf occlusion, and small disease spots. Thus, the robustness of the model in these challenging scenarios has not been fully validated experimentally. Second, the visual similarity between different disease categories reveals room for improvement in the discrimination of subtle features. Third, this study currently covers only four types of bean diseases, leaving the generalizability to a wider range of disease types (such as anthracnose and bacterial spot) unclear. Finally, the model’s ability to classify disease severity remains unexplored, which is critical for precise pesticide application and postharvest quality assessment in actual agricultural production.

To address the above limitations, future research can be advanced in the following directions. First, construct a bean leaf disease image dataset that includes multiple challenging conditions, such as varying lighting, occlusion, complex backgrounds, and small disease spots, and design targeted experiments to quantitatively evaluate model robustness. Second, introduce more sophisticated attention mechanisms or metric learning strategies to strengthen the model’s ability to distinguish between diseases with similar visual features. Third, expand the coverage of disease categories and enhance generalization ability through few-shot learning or domain adaptation techniques. Fourth, explore multilevel classification tasks for disease severity and analyze disease development patterns based on time-series data. In addition, we plan to further optimize the model structure for lighter edge deployment, such as integration with drones or mobile devices, to provide intelligent solutions for real-time monitoring and postharvest quality control of bean diseases.

## 5 Conclusions

In this work, we propose a novel deep learning framework that overcomes key limitations of conventional models by integrating lightweight architecture design with intelligent feature enhancement. Through systematic analysis of disease characteristics, we structurally reformulated the classical ZFNet, reducing redundant parameters while significantly improving feature extraction efficiency. Our approach pioneers the deep embedding of a dual attention mechanism that simultaneously processes channel and spatial dimensions, enabling adaptive focus on critical pathological traits and substantially enriching feature discriminability. Additionally, we incorporated residual modules to establish efficient gradient propagation pathways, accelerating convergence while improving accuracy.

The proposed model demonstrates superior performance compared to mainstream lightweight architectures, maintaining competitive parameter counts suitable for edge deployment while achieving reduced computational complexity and faster inference. Our results surpass those reported in recent plant pathology studies, setting a new benchmark for crop disease identification, with direct applicability to staple grains. This research provides a practical solution for intelligent disease management in agronomic production and postharvest quality preservation, particularly for key crops where early pathogen detection is essential for minimizing postharvest losses, maintaining food safety, and protecting yield.

Despite these advances, several challenges require further investigation to enhance practical implementation. First, future work will expand datasets to cover diverse geographical regions and crop diseases, including major rice pathogens, with images collected under varying paddy field and postharvest storage conditions to improve generalizability. Beyond classification, we will employ deep learning techniques to analyze disease severity through lesion quantification, implement image enhancement algorithms, and develop metrics for assessing identification difficulty across pathological manifestations and their impact on product quality. Second, to address practical monitoring needs from field to storage, we will advance from static image analysis to real-time video processing, establishing continuous surveillance capabilities for automated disease identification and data logging in crop cultivation and postharvest handling areas. Third, we will develop dedicated mobile applications compatible with smartphones and tablets, integrating camera capabilities with augmented reality to support field and postharvest diagnosis and visual interaction with disease information. Through these developments, we aim to enhance model adaptability, real-time performance, and practical value across diverse agronomic and postharvest environments, ultimately contributing to more sustainable crop cultivation and food supply systems. The recognition performance of the model in highly variable scenarios, such as field lighting changes and leaf overlap, still requires further validation. Field lighting conditions vary dramatically, including direct sunlight, shade from trees, scattered light on cloudy days, and low light at dawn and dusk. These variations can significantly alter leaf color, brightness, and texture features, yet the current model lacks a systematic evaluation of feature consistency under different lighting conditions. Moreover, leaf occlusion is common within crop canopies, where disease spots may be partially or completely covered by healthy upper leaves or overlap with veins and leaf edges. This poses a potential challenge to the model’s ability to discriminate based on local lesion features. During low-altitude acquisition by drones, issues such as leaf motion blur, inconsistent image resolution, and geometric distortion due to varying shooting angles may interfere with accurate localization and classification of lesion areas. Therefore, further targeted validation and optimization are needed to ensure reliable transfer of the current model’s performance from standard datasets to actual drone inspection scenarios.

## Data Availability

The original contributions presented in the study are included in the article/supplementary material. Further inquiries can be directed to the corresponding authors.
